# First-principles prediction of ferromagnetic half-Heusler LiMnTe for spintronic applications

**DOI:** 10.1039/d6ra06313f

**Published:** 2026-09-01

**Authors:** Thi H. Ho

**Affiliations:** a Laboratory for Computational Physics, Institute for Computational Science and Artificial Intelligence, Van Lang University Ho Chi Minh City Vietnam thi.hohuynh@vlu.edu.vn; b Faculty of Mechanical, Electrical, and Computer Engineering, Van Lang School of Technology, Van Lang University Ho Chi Minh City Vietnam

## Abstract

First-principles calculations were performed to investigate the structural stability, magnetism, electronic structure, and intrinsic hall transport of half-Heusler LiMnTe. Among the considered phases, ferromagnetic β-LiMnTe is identified as the ground-state configuration, with an equilibrium lattice constant of 6.39 Å and an integer magnetic moment of 4.00 *µ*_B_ per f.u. Its energetic, mechanical, and dynamical stability is supported by the negative formation energy, satisfaction of the Born–Huang criteria, and absence of imaginary phonon modes. At the PBE level, the Mn-d-dominated electronic structure yields 99.68% spin polarization and nearly half-metallic behavior, whereas DFT + *U* largely preserves the total magnetic moment but reduces the polarization at larger *U*_eff_. Strong nearest-neighbor Mn–Mn exchange stabilizes ferromagnetic ordering and yields a predicted Curie temperature of 352 K. Spin–orbit-coupled Berry-curvature and spin-Berry-curvature hot spots near the Fermi level produce sizable anomalous and spin Hall conductivities of −683.61 S cm^−1^ and −322.35 (*ħ*/*e*) S cm^−1^, respectively. Strain further tunes the spin polarization and Hall responses while preserving ferromagnetism. These results identify β-LiMnTe as a promising candidate for spintronic applications.

## Abstract 2

First-principles calculations predict half-Heusler LiMnTe to be structurally stable and ferromagnetic with near-perfect spin polarization and sizable intrinsic anomalous and spin Hall conductivities.

## Graphical abstract

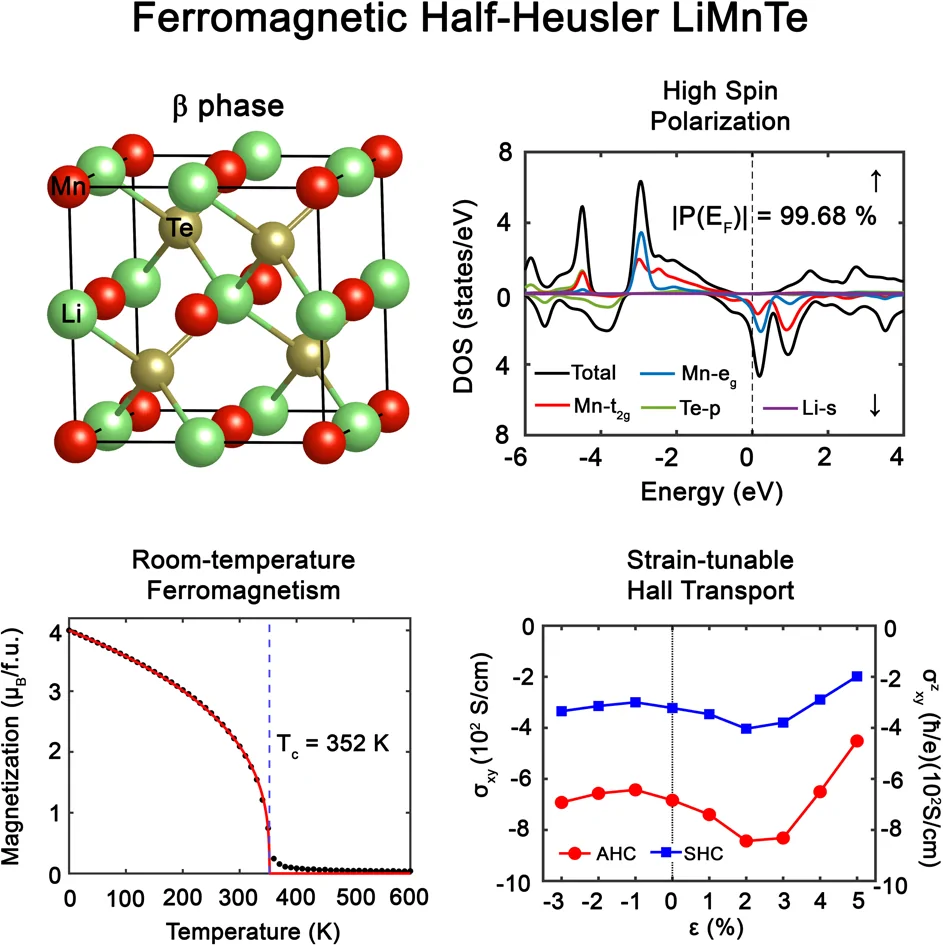

## 1 Introduction

Heusler compounds are a versatile class of functional materials for spintronic, thermoelectric, topological, and magneto-electronic applications because their electronic and magnetic properties can be widely tuned through chemical substitution.^1–5^ Among them, half-Heusler compounds with the general formula *XYZ* and the *C*1_*b*_ structure are particularly attractive because of their chemical flexibility, broken inversion symmetry, and close relationship to binary semiconductors.^6,7^ Since the discovery of half-metallic ferromagnetism in NiMnSb, these materials have been widely investigated as potential spin injectors and spin filters.^3,8,9^ Their spin-selective gaps and integer magnetic moments are often described by Slater–Pauling electron-counting rules, providing a useful guide for designing highly spin-polarized materials.^10,11^

First-principles screening has substantially expanded the compositional space of half-Heuslers, revealing semiconducting, half-metallic, and nearly half-metallic candidates, although thermodynamic stability remains essential for assessing their experimental feasibility.^12–14^ In this context, alkali-metal-based half-Heuslers have attracted increasing attention because charge donation from the electropositive alkali atom can modify the electron count, exchange splitting, magnetic moment, and spin-dependent band structure.^15–19^ In particular, LiMnZ compounds have been predicted to exhibit large magnetic moments and half-metallic behavior under suitable structural conditions.^16^

Li-based telluride half-Heuslers, however, remain less explored than their pnictide- and lighter-chalcogen-based counterparts. Li can strongly influence electron counting and magnetic exchange, whereas the heavy Te atom introduces substantial spin–orbit coupling and may promote Berry-curvature-driven phenomena, including anomalous Hall, spin Hall, and topological transport.^7,15,18,20^ The predicted spin-polarized Weyl states in KCrTe and RbCrTe further demonstrate the potential of alkali-transition-metal telluride half-Heuslers for combining strong spin polarization with nontrivial transport.^21^ Nevertheless, cubic half-Heusler LiMnTe has not been systematically examined, while previous studies of the Li–Mn–Te system have mainly focused on layered LiMnTe_2_.^22,23^

In this work, we present a first-principles investigation of the structural, thermodynamic, mechanical, dynamical, magnetic, electronic, and spin-transport properties of half-Heusler LiMnTe. Different atomic arrangements and magnetic configurations are compared to identify the ground-state structure. The origin and robustness of its spin-polarized electronic structure are analyzed in terms of Mn-d and Te-p hybridization, and strain effects. Finally, spin–orbit-coupled Berry-curvature calculations are used to evaluate the intrinsic anomalous Hall and spin Hall conductivities, providing a unified assessment of LiMnTe as a potential material for high-spin-polarization and spin-transport applications.

## 2 Computational methods

All first-principles calculations were performed within density functional theory (DFT) using the all-electron full-potential linearized augmented-plane-wave (FLAPW) method implemented in the FLEUR code.^24,25^ The exchange–correlation interaction was treated using the generalized gradient approximation in the Perdew–Burke–Ernzerhof (PBE) parametrization.^26^ The muffin-tin radii, *R*_MT_, were set to 1.80, 2.20, and 2.35 a.u. for Li, Mn, and Te, respectively, ensuring that the muffin-tin spheres remained non-overlapping for all optimized structures. The plane-wave basis cutoff was defined by *R*^min^_MT_*K*_max_ = 8.0, while the cutoff for the charge-density and potential expansions was set to *G*_max_ = 13.3 a.u.^−1^. The angular-momentum expansion inside the muffin-tin spheres was truncated at *l*_max_ = 8. Brillouin-zone integrations were carried out using an 8 × 8 × 8 ***k***-point mesh for structural optimization and a denser 15 × 15 × 15 mesh for accurate total-energy and electronic density-of-states calculations. The self-consistent-field cycle was considered converged when the change in total energy between successive iterations was smaller than 10^−6^ eV. Spin-unpolarized calculations were performed for the nonmagnetic state, whereas spin-polarized calculations were employed for the ferromagnetic and antiferromagnetic configurations. Spin–orbit coupling (SOC) was included in the Berry-curvature calculations used to evaluate the intrinsic anomalous Hall and spin Hall conductivities. To assess the influence of on-site Coulomb correlations on the Mn-3d states, additional DFT + *U* calculations were performed following the FLAPW implementation,^27^ with *U*_eff_ varied from 0 to 5 eV.

**Fig. 1:**
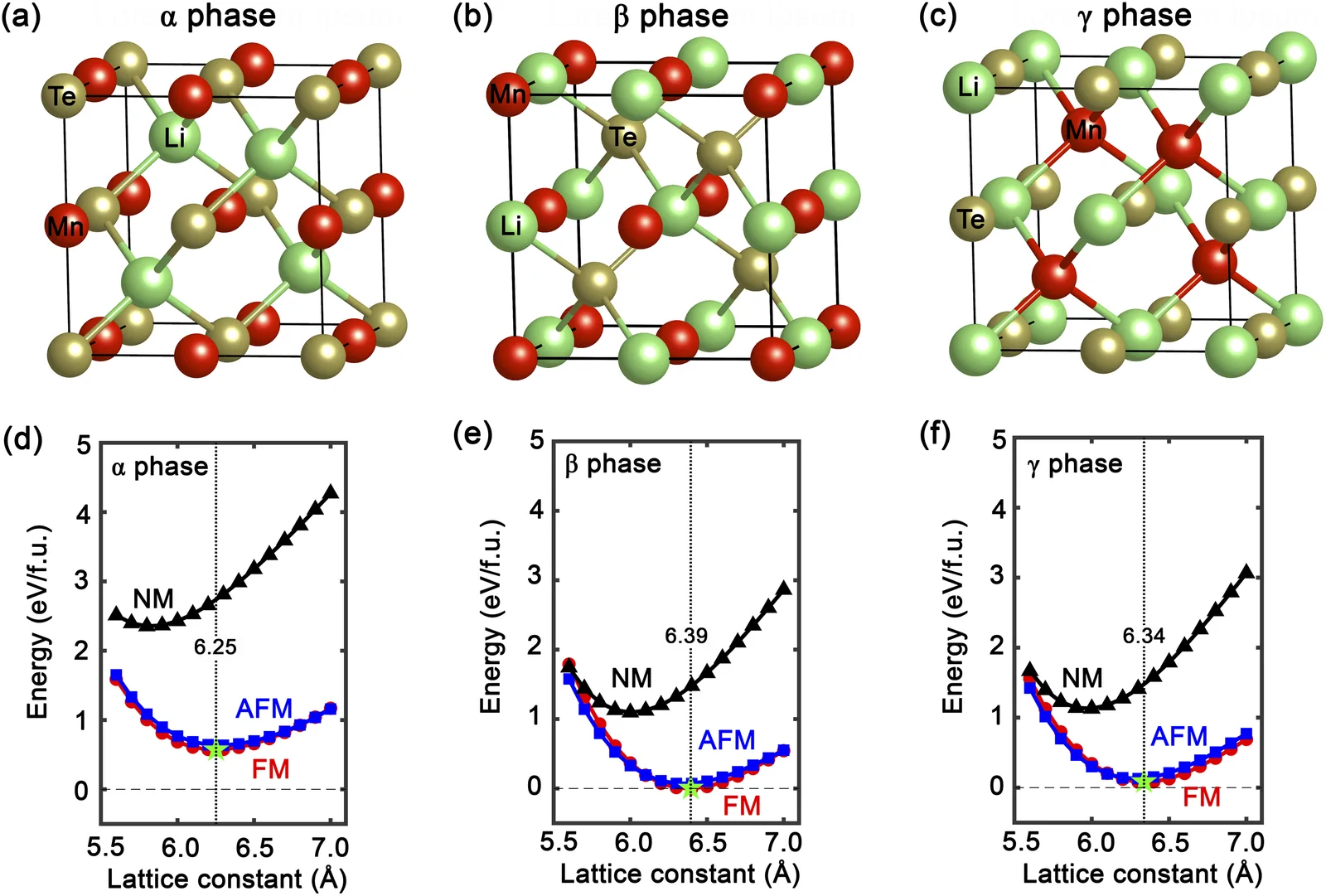
Crystal structures and magnetic energetics of half-Heusler LiMnTe. (a–c) The *C*1_*b*_ crystal structures of the α-, β-, and γ-phase LiMnTe compounds, respectively. Li, Mn, and Te atoms are shown in green, red, and gold, respectively. (d–f) Total-energy variation as a function of lattice constant for the α-, β-, and γ phases under non-magnetic (NM, black), antiferromagnetic (AFM, blue), and ferromagnetic (FM, red) configurations. The vertical dashed lines indicate the equilibrium lattice constants, and the energies are referenced to the lowest-energy configuration.

LiMnTe was modeled in the cubic half-Heusler (*C*1_*b*_) structure with space group *F*4̄3*m* (No. 216). This structure can be viewed as a regular full-Heusler (*L*2_1_) lattice in which one of the four interpenetrating fcc sublattices is vacant.^2,6,18^ Three distinct atomic arrangements, denoted as the α, β, and γ phases, are illustrated in Fig. 1(a–c). These configurations were generated by distributing Li, Mn, and Te over the occupied Wyckoff positions 4*a* (0, 0, 0), 4*b* (1/2, 1/2, 1/2), and 4*c* (1/4, 1/4, 1/4), while keeping the 4*d* (3/4, 3/4, 3/4) position vacant. In the α phase, Mn and Te form a rocksalt-type framework, with Li occupying half of the tetrahedral interstitial sites. In the β phase, Li and Mn form the rocksalt-type sublattice, while Te occupies the tetrahedral site. Equivalently, this arrangement may be regarded as a zinc-blende-like MnTe network interpenetrated by an fcc Li sublattice. In the γ phase, Li and Te form the rocksalt-type framework with Mn occupying the tetrahedral site.

The relative stability of the three phases and their possible magnetic configurations was first determined from total-energy calculations as a function of lattice constant. The thermodynamic stability of the lowest-energy phase was subsequently evaluated from its formation energy, *E*_form_. Its mechanical and dynamical stabilities were examined through the calculated elastic constants and phonon dispersion relations, respectively. The structure satisfying the energetic, thermodynamic, mechanical, and dynamical stability criteria was then selected for detailed analyses of its electronic structure, magnetic properties, exchange interactions, finite-temperature magnetic behavior, and intrinsic anomalous and spin Hall conductivities. Additional computational details are provided in the SI.

## 3 Results and discussion

### 3.1 Structural properties

To determine the equilibrium structure and magnetic ground state of LiMnTe, total energies were calculated as a function of lattice constant for the nonmagnetic (NM), ferromagnetic (FM), and antiferromagnetic (AFM) states of the α, β, and γ phases, as illustrated in Fig. 1(d)–(f). In all three phases, the NM state is considerably higher in energy than the spin-polarized states, indicating the importance of magnetism in stabilizing LiMnTe. Among the configurations considered, the β phase exhibits the lowest-energy minimum. A magnified comparison of the low-lying FM and AFM states in Fig. S1 further confirms that the FM β phase is the ground-state configuration of cubic LiMnTe half-Heusler.

The equilibrium properties of β-LiMnTe are summarized in Table 1. The optimized lattice constant is 6.39 Å, and the energy difference Δ*E* = *E*_AFM_ − *E*_FM_ is 68.10 meV per f.u., confirming the energetic preference for ferromagnetic ordering. The magnetic moment is mainly localized on Mn, with a value of 3.69 *µ*_B_, whereas Li and Te carry negligible moments of 0.00 and 0.02 *µ*_B_, respectively. The total moment is 4.00 *µ*_B_ per f.u., consistent with Slater–Pauling-type electron counting in half-metallic half-Heusler compounds^10,16^ and indicative of a strongly spin-polarized electronic structure.

**Table 1:** Calculated equilibrium properties of LiMnTe in the β phase, including the energy difference Δ*E* = *E*_AFM_ − *E*_FM_, lattice constant *a*, atom-projected magnetic moments *m*_i_, total magnetic moment *m*_tot_, and formation energy *E*_form_

Δ*E* (meV per f.u.)	*a* (Å)	*m*_Li_ (*µ*_B_)	*m*_Mn_ (*µ*_B_)	*m*_Te_ (*µ*_B_)	*m*_tot_ (*µ*_B_)	*E*_form_ (eV per f.u.)
68.10	6.39	0.00	3.69	0.02	4.00	−0.57

The calculated moment is comparable to that reported for β-LiMnSi and follows the general trend of LiMnZ and alkali-metal XCrZ half-Heuslers, in which magnetism is dominated by transition-metal d states.^16,17^ In contrast to LiMnN and LiMnP, for which AFM ordering was predicted to be favorable, LiMnTe stabilizes the FM state. This difference suggests that replacing lighter main-group elements with Te modifies the exchange interactions and promotes ferromagnetism in the β phase.

### 3.2 Structural stability

The thermodynamic stability of β-LiMnTe was evaluated from its formation energy relative to decomposition into bcc Li and zinc blende (ZB) MnTe, calculated as follows:^18^

**Fig. 2:**
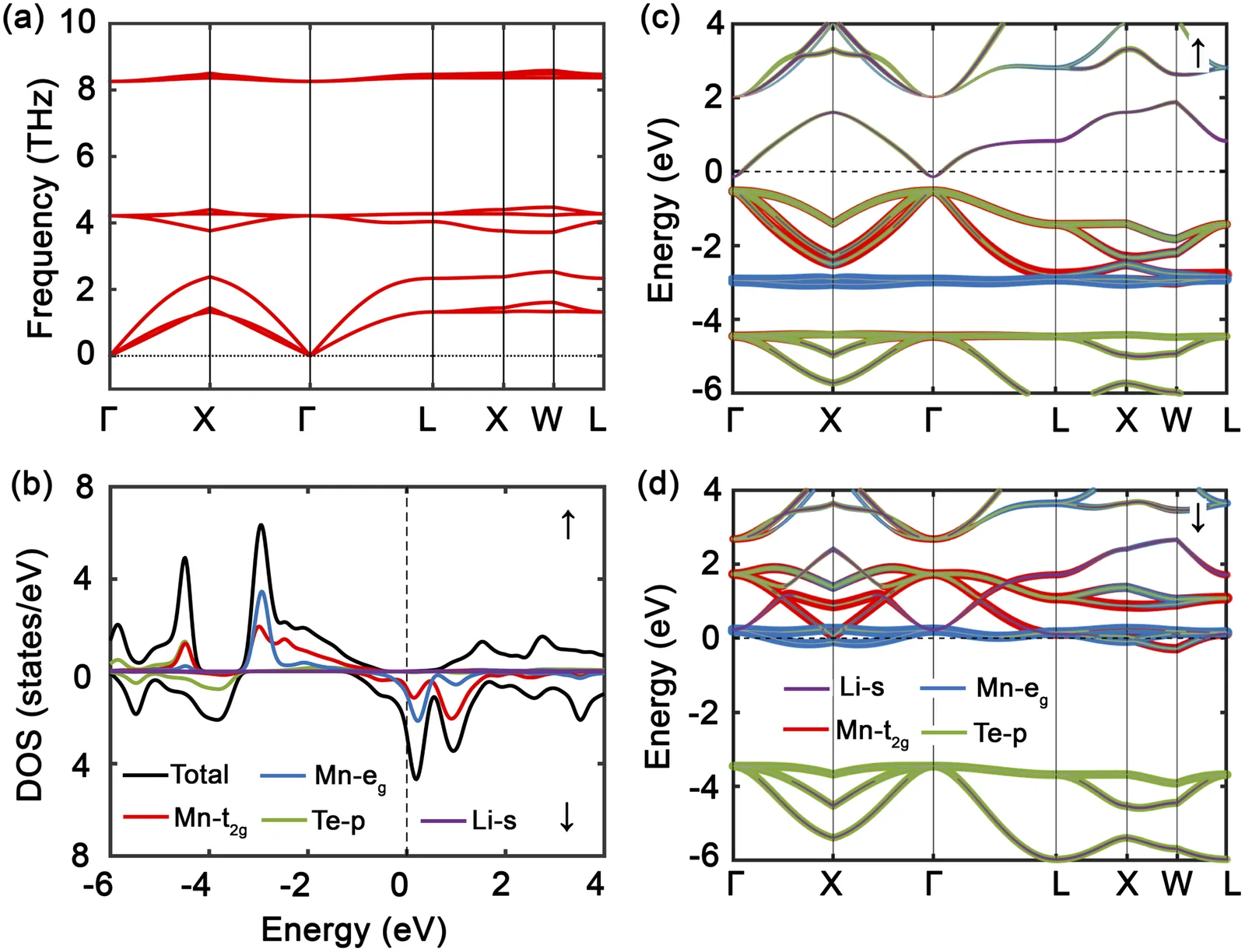
Phonon dispersion, spin-resolved orbital-projected density of states, and spin-resolved band structures of ferromagnetic β-LiMnTe. (a) Phonon spectrum along the high-symmetry path. (b) Spin-resolved total and orbital-projected DOS for Li-s, Mn-e_g_, Mn-*t*_2g_, and Te-p states. (c) Spin-up and (d) spin-down orbital-projected band structures along the same high-symmetry path. The colors indicate the dominant orbital contributions, and the Fermi level is set to zero.

1 *E*_form_ = *E*_LiMnTe_ − (*E*_Li_ + *E*_MnTe_)

where *E*_LiMnTe_, *E*_Li_, *E*_MnTe_ are total energies of LiMnTe, bcc Li, and ZB-MnTe, respectively. As shown in Table 1, *E*_form_ = −0.57 eV per f.u. indicates that the formation of β-LiMnTe is energetically stable against decomposition into bcc Li and ZB-MnTe. The phonon dispersion of the stable ferromagnetic β-LiMnTe phase is shown in Fig. 2(a). The absence of imaginary phonon frequencies throughout the Brillouin zone confirms the dynamical stability of the optimized structure, demonstrating that ferromagnetic β-LiMnTe is dynamically stable.

The calculated elastic constants and mechanical parameters of ferromagnetic β-LiMnTe are summarized in Table 2. The elastic constants *C*_11_, *C*_12_, and *C*_44_ are 67.72, 37.50, and 36.25 GPa, respectively. For a cubic crystal, the Born–Huang mechanical stability criteria are expressed as,^28,29^

2 *C*_11_ − *C*_12_ > 0, *C*_11_ + 2*C*_12_ > 0, *C*_44_ > 0.

**Table 2:** Computed elastic constants (*C*_*ij*_), bulk modulus (*B*), shear modulus (*G*), Young's modulus (*E*), Poisson's ratio (*ν*), anisotropy factor (*A*), and Pugh's ratio (*B*/*G*) of ferromagnetic β-LiMnTe

*C*_11_	*C*_12_	*C*_44_	*B*	*G*	*E*	*ν*	*A*	*B*/*G*
(GPa)	(GPa)	(GPa)	(GPa)	(GPa)	(GPa)			
67.72	37.50	36.25	47.57	25.52	64.94	0.27	2.40	1.86

For β-LiMnTe, these conditions are fully satisfied, with *C*_11_ − *C*_12_ = 30.22 GPa, *C*_11_ + 2*C*_12_ = 142.72 GPa, and *C*_44_ = 36.25 GPa. This confirms that ferromagnetic β-LiMnTe is mechanically stable. In addition, the bulk modulus *B* is calculated to be 47.57 GPa, indicating a moderate resistance to volume compression. The shear modulus *G* and Young's modulus *E* are 25.52 and 64.94 GPa, respectively, which are lower than those of many conventional transition metals and transition-metal carbides and nitrides,^30,31^ suggesting that β-LiMnTe is relatively soft. The Poisson's ratio *ν* is 0.272, which is within the typical range for mechanically stable crystalline solids.^32^ In addition, the Pugh's ratio *B*/*G* is 1.86, larger than the critical value of 1.75,^33^ suggesting a ductile mechanical character. The elastic anisotropy factor *A* is 2.40, deviating from unity and indicating elastic anisotropy in β-LiMnTe. Therefore, the elastic-constant analysis confirms the mechanical stability of ferromagnetic β-LiMnTe and provides further support for its structural stability.

Previous studies of the Li–Mn–Te system have mainly reported layered LiMnTe_2_, which represents a plausible competing phase during the synthesis of β-LiMnTe, particularly under Te-rich conditions. Although LiMnTe_2_ has a different stoichiometry and is therefore not a polymorph of LiMnTe, their thermodynamic competition can be assessed using the following compositionally balanced reaction:

3 LiMnTe + Te → LiMnTe_2_.

The corresponding reaction energy was calculated as

4 Δ*E*_rxn_ = *E*_LiMnTe_2__ − *E*_LiMnTe_ − *E*_Te_,

where *E*_LiMnTe_2__ and *E*_LiMnTe_ are the total energies per formula unit of layered LiMnTe_2_ and β-LiMnTe, respectively, and *E*_Te_ is the energy per atom of bulk elemental Te, corresponding to the Te-rich limit. The calculated value, Δ*E*_rxn_ = −0.67 eV per reaction, indicates that the formation of layered LiMnTe_2_ from β-LiMnTe and Te is energetically favorable under Te-rich conditions. Thus, although β-LiMnTe is energetically favorable against decomposition into bcc Li and ZB-MnTe and is mechanically and dynamically stable, it remains metastable with respect to tellurization into layered LiMnTe_2_.

Although β-LiMnTe has not yet been synthesized, its mechanical and dynamical stability suggests that it may be experimentally accessible as a kinetically stabilized phase, despite the thermodynamic driving force toward layered LiMnTe_2_ under Te-rich conditions. Experimental studies of related compounds suggest two possible synthesis routes. For bulk synthesis, air-sensitive layered LiMnTe_2_ has been prepared under inert conditions,^22^ while the related 1 : 1 : 1 compound LiMnAs was synthesized from its constituent elements in an Ar-sealed Ta ampoule and exhibited a high-temperature cubic MgAgAs-type phase.^34^ These precedents suggest that bulk β-LiMnTe could be explored by annealing a glovebox-prepared 1 : 1 : 1 mixture in a sealed refractory-metal ampoule, while avoiding excess Te, adjusting the Li loading to compensate for Li volatility, and employing rapid cooling or quenching to suppress layered LiMnTe_2_ formation and kinetically retain the cubic phase. Given its predicted metastability, nonequilibrium thin-film growth may offer a more promising route. The successful growth of *C*1_*b*_ CuMnSb and NiMnSb films by molecular-beam epitaxy and magnetron co-sputtering, respectively,^35,36^ suggests that controlled Li–Mn–Te codeposition on a lattice-matched substrate could enable the kinetic or epitaxial stabilization of β-LiMnTe.

### 3.3 Electronic structure

The spin-resolved density of states (DOS) of ferromagnetic β-LiMnTe is shown in Fig. 2(b). A large spin asymmetry occurs near the Fermi level (*E*_F_), where the spin-down states dominate and the spin-up contribution is strongly suppressed, yielding a spin polarization of |*P*(*E*_F_)| = 99.68%. The slight deviation from the ideal value of 100% originates from a small but finite spin-up DOS at *E*_F_, which lies close to the edge of the spin-up pseudogap, while several Mn d-derived spin-down bands cross *E*_F_, as shown in Fig. 2(c) and (d). Consequently, the DOS at *E*_F_ is dominated by the spin-down channel, indicating nearly half-metallic behavior. The states near *E*_F_ are primarily derived from exchange-split Mn-d orbitals, with appreciable Mn-d and Te-p hybridization in the occupied region and only a weak Li-s contribution. These results demonstrate that Mn governs the electronic and magnetic properties, whereas Li mainly acts as an electron donor. Strain engineering may shift the spin-up band edge away from *E*_F_ and enhance the polarization toward the ideal half-metallic limit,^16,37^ as discussed further in Section 3.6.

### 3.4 Exchange coupling and Curie temperature

**Fig. 3:**
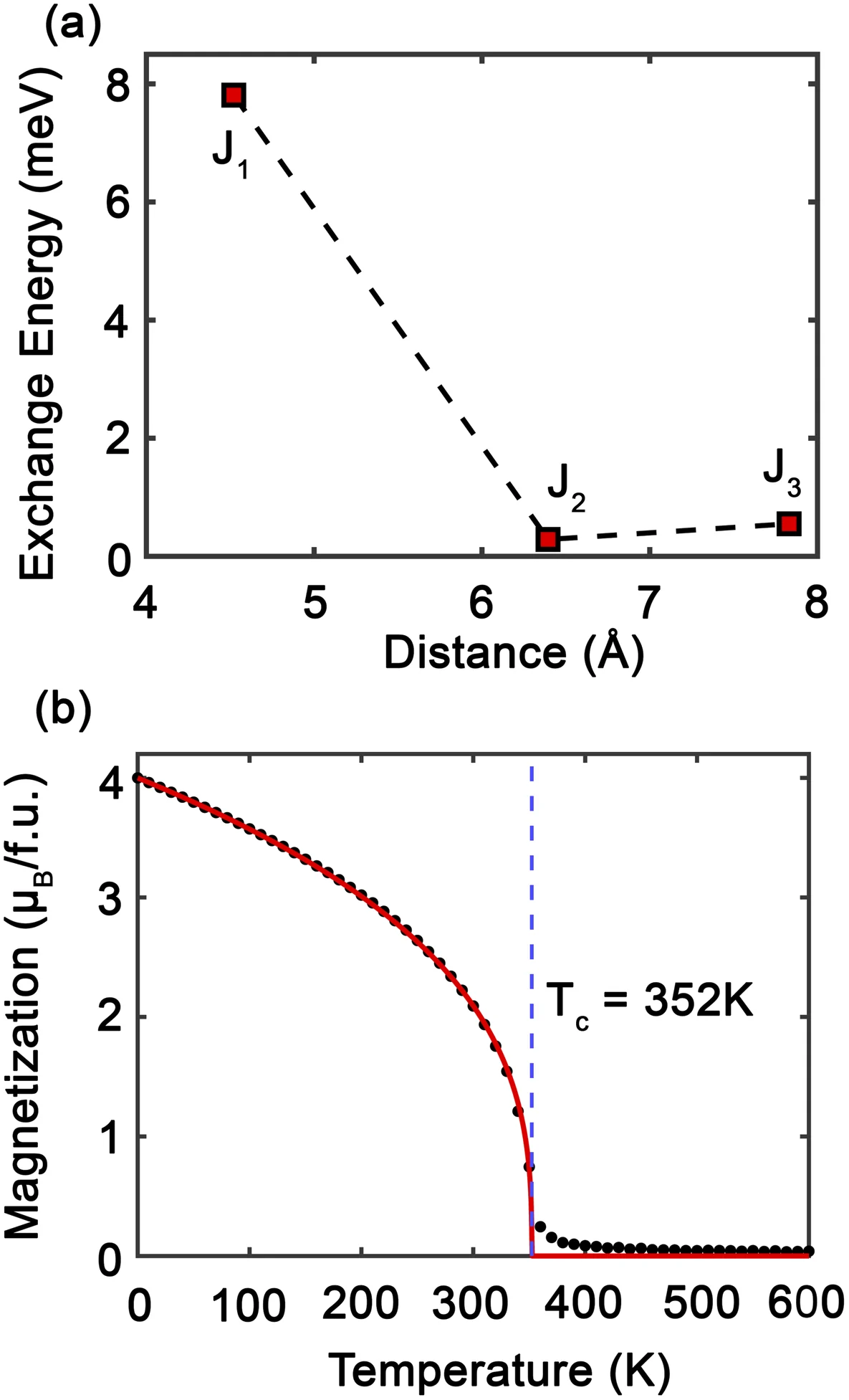
Magnetic exchange interactions and Curie temperature of ferromagnetic β-LiMnTe. (a) Calculated exchange energies as a function of Mn–Mn distance, where *J*_1_, *J*_2_, and *J*_3_ denote the first-, second-, and third-nearest-neighbor exchange interactions, respectively. (b) Temperature-dependent magnetization obtained from atomistic Monte Carlo simulation. The black points represent simulation results, while the solid line is the critical power-law fit. The Curie temperature is estimated to be *T*_C_ = 352 K, as indicated by the vertical dashed line.

To clarify the microscopic origin and thermal stability of ferromagnetism in β-LiMnTe, the Mn–Mn exchange interactions were analyzed. Fig. 3(a) shows that the nearest-neighbor Mn–Mn exchange interaction dominates in β-LiMnTe, with *J*_1_ = 7.81 meV at 4.52 Å, whereas *J*_2_ = 0.29 meV and *J*_3_ = 0.55 meV are much weaker. This confirms that the ferromagnetic ground state is mainly stabilized by short-range Mn–Mn exchange coupling. From the atomistic Monte Carlo simulation, the temperature-dependent magnetization in Fig. 3(b) decreases from approximately 4.00 *µ*_B_ per f.u. and vanishes near the predicted Curie temperature of 352 K. The predicted *T*_C_ should be regarded as an estimate because the present model neglects magnetic anisotropy, longitudinal spin fluctuations, and quantum-spin effects, all of which may influence the transition temperature. However, the calculated *T*_C_ suggests that ferromagnetic ordering can persist above room temperature. Its value is comparable to values reported for several alkali-metal-based half-Heuslers, which typically lie in the range of 300–450 K.^38^

To assess the finite-temperature structural stability of β-LiMnTe near its predicted Curie temperature, spin-polarized *ab initio* molecular dynamics simulations were performed at 350 K. As shown in Fig. S8 (See SI), the temperature fluctuated around the target value, while the total energy remained bounded throughout the trajectory. The overall half-Heusler framework was preserved. However, the final configuration displayed noticeable displacements of the Mn atoms from their ideal lattice sites. This thermally induced distortion of the Mn sublattice was accompanied by a modest reduction in the total magnetic moment, consistent with a weakening of the ferromagnetic state near the predicted *T*_C_ in Fig. 3(b). These observations support the structural robustness of β-LiMnTe near *T*_C_, while revealing the thermal sensitivity of its Mn sublattice and magnetic response.

### 3.5 Hall transport properties

The intrinsic anomalous Hall conductivity (AHC) and spin Hall conductivity (SHC) of ferromagnetic β-LiMnTe were evaluated using Wannier-interpolated Berry-curvature calculations. The close agreement between the DFT and Wannier-interpolated band structures, shown in Fig. S2 (see SI), confirms the reliability of the constructed Wannier Hamiltonian. At *E*_F_, the calculated AHC and SHC are *σ*_*xy*_ = −683.61 S cm^−1^ and *σ*_*xy*_^*z*^ = −322.35 (*ħ*/*e*) S cm^−1^, respectively, indicating sizable intrinsic charge and spin Hall responses.

The magnitude of the AHC, |*σ*_*xy*_| = 683.61 S cm^−1^, is comparable to the calculated intrinsic AHC of bcc Fe, approximately 750.80 S cm^−1^.^39^ It is also larger than the experimental value reported for the half-Heusler compound NiMnSb, about 180 S cm^−1^ at 2 K, although smaller than the much larger value of approximately 2200 S cm^−1^ measured for PtMnSb.^40^ Compared with full-Heusler compounds, the AHC of β-LiMnTe remains below the values reported for Rh_2_MnAl, Co_2_MnAl, Co_2_MnGa, and Cu_2_CoSn, which range from approximately 1119 to 1649 S cm^−1^.^41^ Nevertheless, the calculated value is substantial for a Li-based half-Heusler and places β-LiMnTe among Heusler systems with a finite anomalous Hall response.

The SHC magnitude, |*σ*_*xy*_^*z*^| = 322.35 (*ħ*/*e*) S cm^−1^, is much lower than that of fcc Pt, for which an intrinsic SHC of approximately 2000 (*ħ*/*e*) S cm^−1^ has been reported.^42^ However, it is comparable to the effective SHC range estimated for YPtBi thin films, approximately 140–420 (*ħ*/*e*) S cm^−1^.^43^ Among full-Heusler compounds, the calculated SHC is close to that of Fe_2_NiGa, 311.02 (*ħ*/*e*) S cm^−1^, and considerably larger than that of Co_2_FeAl, 29.60 (*ħ*/*e*) S cm^−1^, although it remains below the values of approximately 579–729 (*ħ*/*e*) S cm^−1^ reported for several high-SHC full-Heusler systems.^41^ These comparisons demonstrate that β-LiMnTe simultaneously supports sizable anomalous and spin Hall conductivities, highlighting its potential for transverse charge transport, spin-current generation, and spintronic applications.

**Fig. 4:**
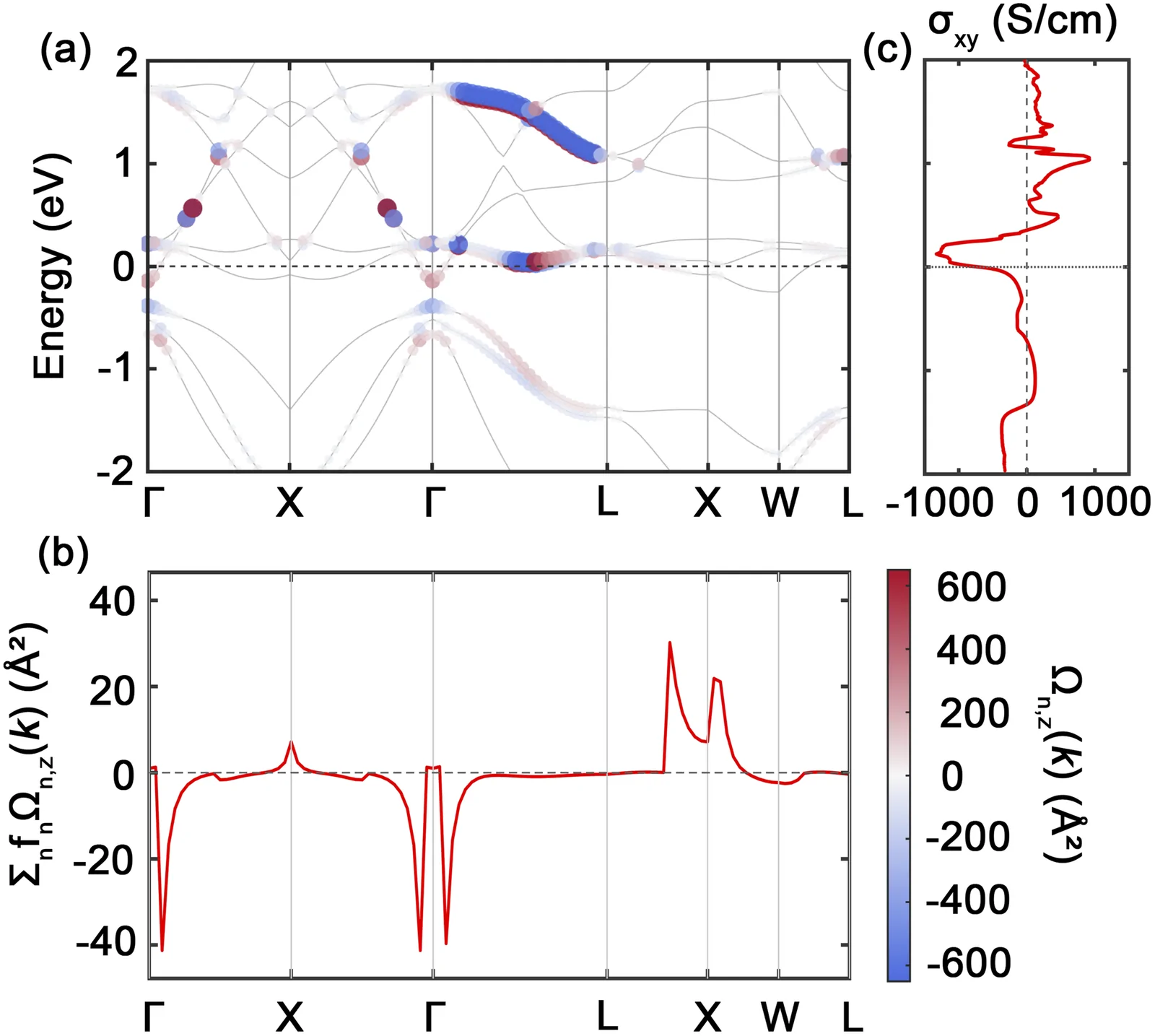
Berry curvature and anomalous Hall conductivity of ferromagnetic β-LiMnTe. (a) Berry-curvature-resolved band structure, where the color scale represents Ω_*n*,*z*_(**k**). (b) ***k***-resolved summed Berry curvature 
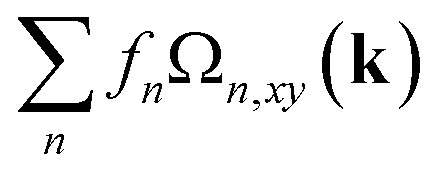
 along the high-symmetry path. (c) Fermi-level-dependent anomalous Hall conductivity *σ*_*xy*_, with the dashed line marking the Fermi level. The Fermi level is set to zero energy.

**Fig. 5:**
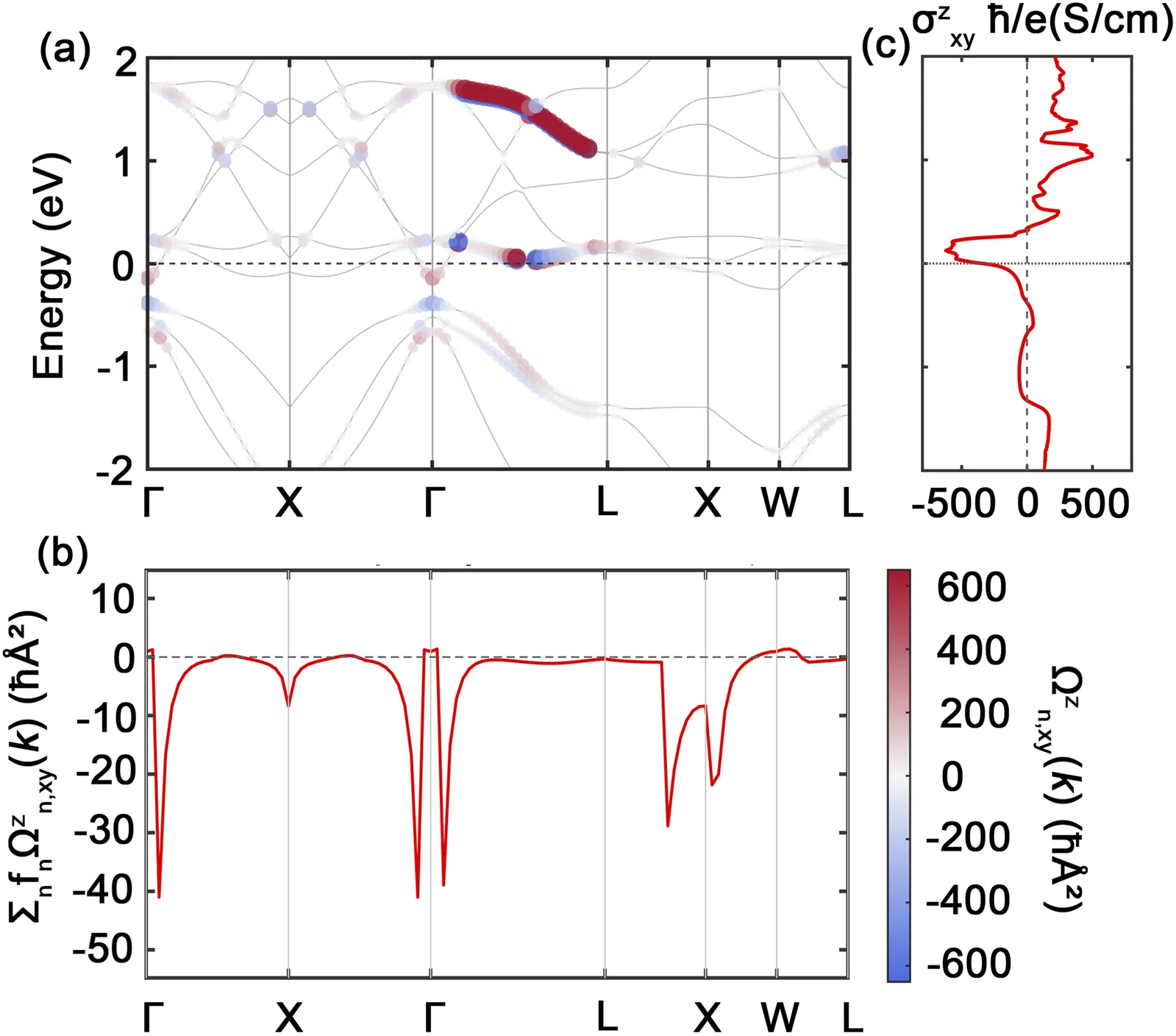
Spin Berry curvature and spin Hall conductivity of ferromagnetic β-LiMnTe. (a) Spin-Berry-curvature-resolved band structure along the high-symmetry path, where the color scale represents *Ω*_*n*,*xy*_^*z*^(**k**). (b) *k*-resolved summed spin Berry curvature 
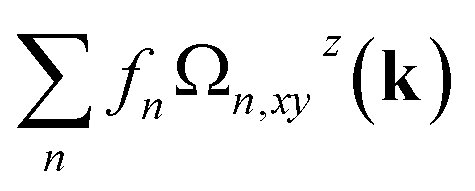
 along the high-symmetry path. (c) Fermi-level-dependent spin Hall conductivity *σ*_*xy*_^*z*^, with the dashed line marking the Fermi level. The Fermi level is set to zero energy.

To elucidate the microscopic origin of AHC and SHC in ferromagnetic β-LiMnTe, the Berry curvature and spin Berry curvature were analyzed along the high-symmetry ***k*** path. Fig. 4 and 5 present the curvature-resolved band structures, the corresponding band-summed ***k***-resolved contributions, and the Hall conductivities as functions of *E*_F_. In both cases, the dominant contributions are concentrated near *E*_F_, particularly around spin–orbit-coupled bands at *E*_F_. Pronounced curvature hot spots appear near *Γ* and along the *X*–*Γ* and *Γ*–*L* paths, indicating that bands close to *E*_F_ play a central role in the intrinsic Hall responses.

The band-summed quantities, 
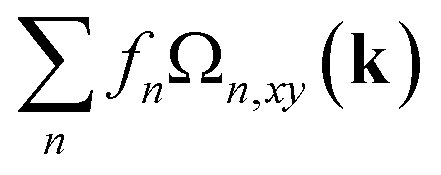
 and 
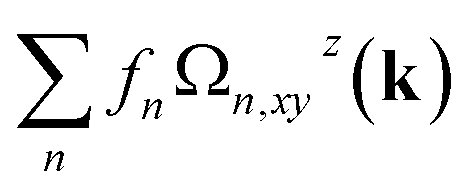
, further reveal strongly localized and sign-changing curvature contributions. For the AHC, large positive and negative peaks occur near *Γ* and along the *L*–*X* and *X*–*W* segments, demonstrating competition between Berry-curvature contributions of opposite signs. The spin Berry curvature is dominated by pronounced negative peaks near *Γ* and along *L*–*X*, consistent with the negative SHC obtained at the intrinsic *E*_F_.

Both conductivities vary strongly with band filling. At the intrinsic *E*_F_, the calculated values are *σ*_*xy*_ = −683.61 S cm^−1^ and *σ*_*xy*_^*z*^ = −322.35 (*ħ*/*e*) S cm^−1^, confirming sizable intrinsic anomalous and spin Hall effects. Shifting *E*_F_ can enhance their magnitudes to approximately −1327 S cm^−1^ and −620 (*ħ*/*e*) S cm^−1^, respectively. These results suggest that carrier doping or electrostatic gating could provide effective routes for tuning and optimizing the Hall responses of β-LiMnTe.

### 3.6 Effects of strain

**Fig. 6:**
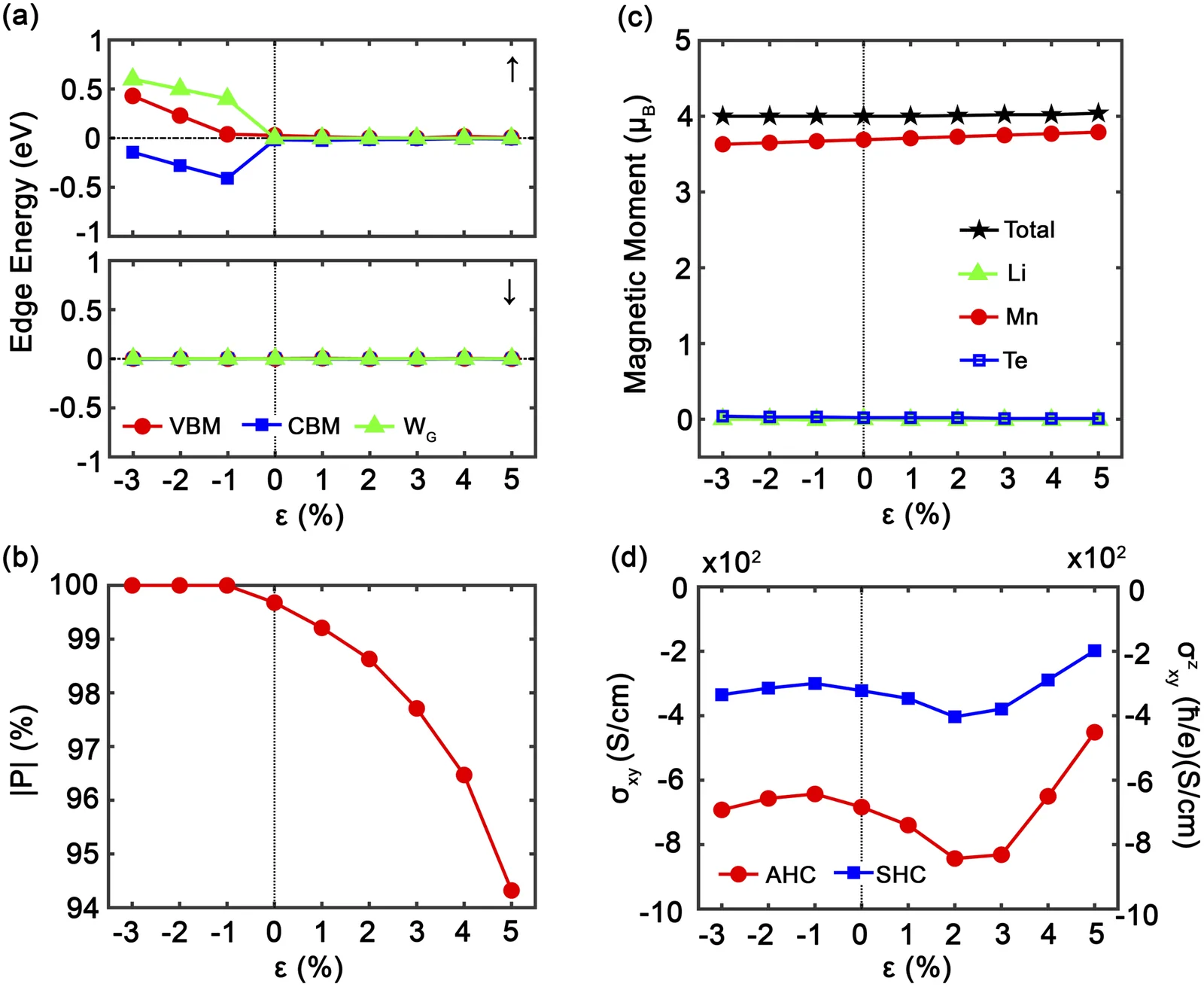
Strain effect on the electronic, magnetic, and Hall transport properties of ferromagnetic β-LiMnTe. (a) Evolution of the valence band maximum (VBM), conduction band minimum (CBM), and width of band gap *W*_G_ for the spin-up and spin-down channels as a function of isotropic volumetric strain. (b) Spin polarization at the Fermi level. (c) Total and atom-projected magnetic moments of Li, Mn, and Te. (d) Strain-dependent anomalous Hall conductivity *σ*_*xy*_ and spin Hall conductivity *σ*_*xy*_^*z*^, respectively. The vertical dotted line indicates the equilibrium state at *ε* = 0%.

The strain effect on β-LiMnTe was investigated to evaluate the robustness and tunability of its spintronic properties. Isotropic volumetric strain ranging from −3% to +5% was applied by uniformly scaling the lattice constant according to *ε* = (*a* − *a*_0_)/*a*_0_ × 100%. It should be noted that the FM ordering remains energetically preferred over the considered AFM configuration throughout this strain range. As shown in Fig. 6(a), compressive strain opens a finite gap in the spin-up channel, whereas tensile strain gradually narrows and nearly closes it. In contrast, the spin-down channel remains metallic over the entire strain range. The microscopic origin of the strain response can be understood from the orbital-projected band structures shown in Fig. S3 and S4 (see SI). In the spin-up channel, compressive strain increases the energetic separation between the occupied Mn-*t*_2g_ and Te-p-dominated states and the lowest unoccupied bands near *E*_F_, thereby opening a finite gap. With increasing tensile strain, these band edges shift toward *E*_F_, progressively reducing and closing the spin-up gap. By contrast, several Mn-d-derived spin-down bands continue to cross *E*_F_ over the entire strain interval, and the spin-down channel therefore remains metallic. Consequently, perfect spin polarization is preserved from −3% to −1%, whereas the increasing spin-up contribution near *E*_F_ under tensile strain reduces |*P*| to approximately 94% at +5% as seen in Fig. 6(b). Despite these band-edge shifts, the exchange splitting and occupation of the Mn-d states remain nearly unchanged, and the total magnetic moment is maintained at 4.00 *µ*_B_ per f.u.

In Fig. 6(d), both AHC and SHC exhibit strain dependence. In particular, the magnitudes of the AHC and SHC are enhanced under tensile strain and reach their maxima near +2% and +3%. This behavior is attributed to the strain-induced shifts of spin–orbit-coupled bands near *E*_F_. As shown in Fig. S5 and S6 (see SI), tensile strain shifts the curvature-carrying bands downward in energy, particularly near the *Γ* point and along the *X*–*Γ* and *Γ*–*L* directions. At strains of approximately +2% to +3%, prominent Berry-curvature and spin-Berry-curvature hot spots move from near or above *E*_F_ to just below *E*_F_. Consequently, these states become occupied and contribute more strongly to the Brillouin-zone integrals that determine the intrinsic AHC and SHC. The resulting redistribution reduces the cancellation between opposite-sign curvature contributions and increases the absolute magnitudes of both conductivities. Thus, tensile strain enhances the Hall responses of β-LiMnTe while preserving its ferromagnetic and highly spin-polarized character.

### 3.7 Effect of on-site Coulomb interactions

**Fig. 7:**
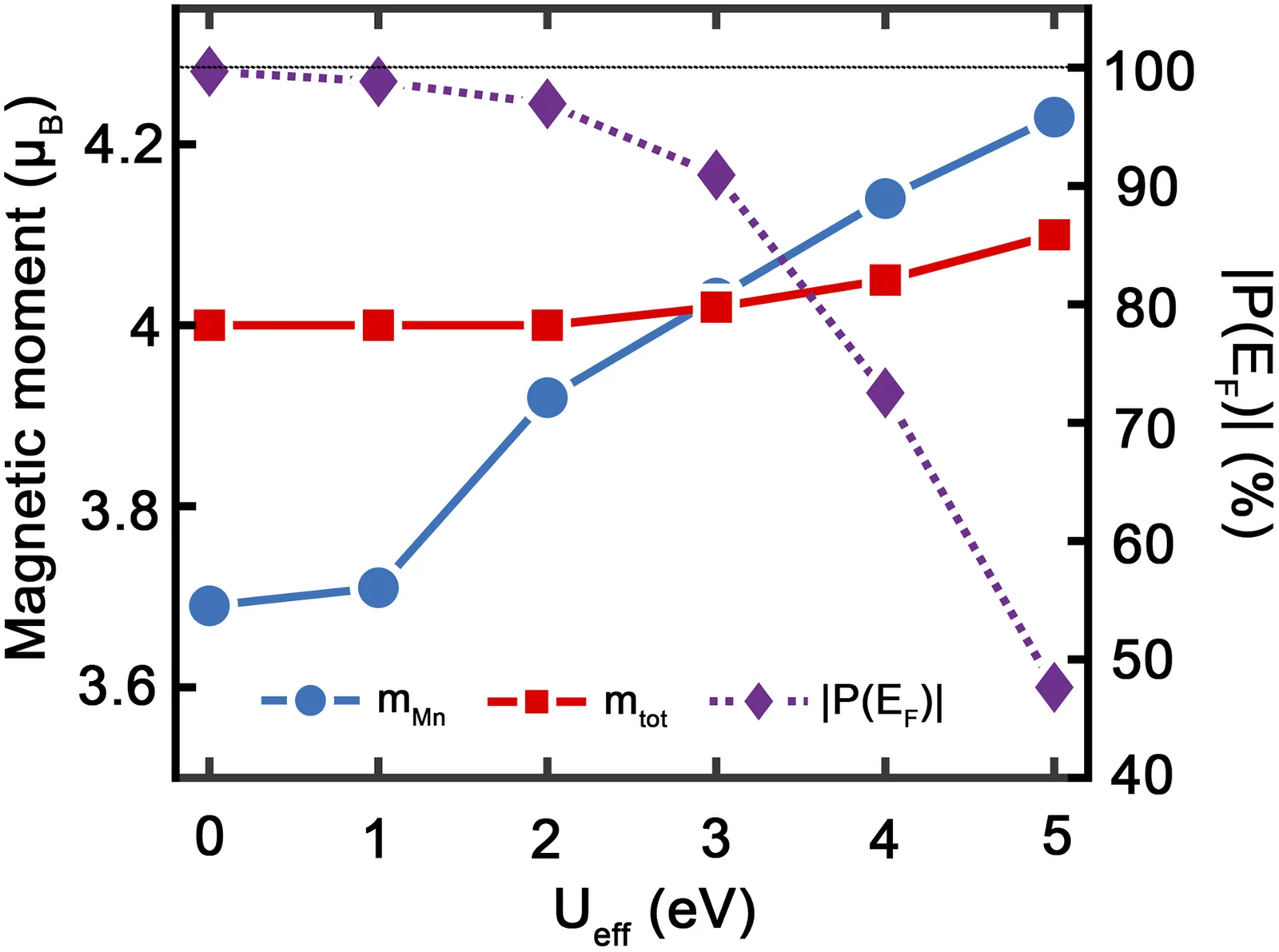
Dependence of the local Mn magnetic moment (*m*_Mn_), total magnetic moment per formula unit (*m*_tot_), and absolute spin polarization at the Fermi level (|*P*(*E*_F_)|) on *U*_eff_ for ferromagnetic β-LiMnTe. The DFT + *U* calculations employ *U*_eff_ = 0 – 5 eV for the Mn-3d orbitals.

Because on-site Coulomb interactions can appreciably modify the electronic structures of Heusler alloys,^44^ the sensitivity of ferromagnetic β-LiMnTe to the Mn-3d interaction was examined using DFT + *U* with *U*_eff_ = 0–5 eV. As shown in Fig. 7, the local Mn moment increases from 3.69 to 4.23 *µ*_B_ as *U*_eff_ increases, whereas the total moment changes only slightly from 4.00 to 4.10 *µ*_B_ per f.u. By contrast, |*P*(*E*_*F*_)| decreases from 99.68% at *U*_eff_ = 0 eV to 96.95%, 90.94%, 72.52%, and 47.64% at *U*_eff_ = 2, 3, 4, and 5 eV, respectively. The corresponding DOS and orbital-resolved band structures shown in Fig. S9–S11 (See SI) reveal a progressive redistribution of the Mn-3d states and changes in the weak spin-up bands near *E*_F_, while the spin-down channel remains metallic. Thus, the nearly half-metallic behavior is maintained approximately for small *U*_eff_ values but becomes increasingly sensitive at larger values.

## 4 Conclusions

First-principles calculations identify ferromagnetic β-LiMnTe as the ground-state structure among the considered half-Heusler phases. The optimized phase has a lattice constant of 6.39 Å and a total magnetic moment of 4.00 *µ*_B_ per f.u., arising predominantly from Mn-d states. Structural stabilities are confirmed by a negative formation energy, the Born–Huang criteria and the absence of imaginary phonon modes.

β-LiMnTe exhibits a strongly spin-asymmetric electronic structure with a spin polarization of 99.68%, indicating nearly half-metallic behavior. Ferromagnetism is mainly stabilized by the nearest-neighbor Mn–Mn exchange interaction, leading to a predicted Curie temperature of 352 K. The compound also shows sizable intrinsic anomalous and spin Hall conductivities of *σ*_*xy*_ = −683.61 S cm^−1^ and *σ*_*xy*_^*z*^ = −322.35 (*ħ*/*e*) S cm^−1^, respectively. These responses originate from significant Berry-curvature hot spots associated with spin–orbit-coupled bands near *E*_F_, particularly around *Γ* and along the *X*–*Γ* and *Γ*–*L* directions. Moreover, strain can tune the spin polarization and Hall conductivities while preserving the ferromagnetic state. These results establish β-LiMnTe as a promising Li-based half-Heusler material combining high spin polarization, a Curie temperature above room temperature, and coupled anomalous and spin Hall transport.

DFT + *U* tests further show that the magnetic moment of the ferromagnetic solution remains comparatively insensitive to *U*_eff_, whereas the Fermi-level spin polarization decreases at larger *U*_eff_. Therefore, the precise degree of half-metallicity and the associated Hall responses should be regarded as quantitatively correlation dependent.

## Author contributions

Thi H. Ho: conceptualization, methodology, validation, formal analysis, investigation, data curation, visualization, writing – original draft, and writing – review and editing.

## Conflicts of interest

There are no conflicts to declare.

## Supplementary Material

RA-016-D6RA06313F-s001

## Data Availability

The data supporting the findings of this study are available from the corresponding author upon reasonable request. Supplementary information (SI): detailed computational methods and additional results on the structural stability, magnetism, electronic structure, and Hall transport of LiMnTe. See DOI: https://doi.org/10.1039/d6ra06313f.
