# Supplementary material for: First-principles prediction of ferromagnetic half-Heusler LiMnTe for spintronic applications
Source: RSC Adv. 2026 Sep 1;16(42):51443–50. doi: 10.1039/d6ra06313f (PMC13531421; doi:10.1039/d6ra06313f)
Supplement: Supplementary file 1 [file RA-016-D6RA06313F-s001.pdf]

Supplementary Information (SI) for RSC Advances.
This journal is © The Royal Society of Chemistry 2026

Supporting Information

First-Principles Prediction of Ferromagnetic Half-Heusler LiMnTe for Spintronic Applications

Thi H. Ho[a,b,*]

[a]Laboratory for Computational Physics, Institute for Computational Science and Artificial Intelligence, Van Lang University, Ho Chi Minh City, Vietnam

[b]Faculty of Mechanical, Electrical, and Computer Engineering, Van Lang School of Technology, Van Lang University, Ho Chi Minh City, Vietnam

[*]Corresponding author

Email: thi.hohuynh@vlu.edu.vn

Detailed Computational Methods

S1. Ferromagnetic and antiferromagnetic configurations

To determine the magnetic ground state of LiMnTe, collinear spin-polarized calculations were performed for ferromagnetic (FM) and antiferromagnetic (AFM) configurations. In the FM configuration, all Mn magnetic moments were initialized parallel to one another. Because the primitive $C1_b$ half-Heusler cell contains only one Mn atom, an AFM arrangement cannot be represented within the primitive cell. Therefore, a 12-atom conventional cell was constructed for AFM calculations. In the AFM configuration, the Mn moments were divided into two magnetic sublattices with equal numbers of spin-up and spin-down Mn atoms.

The relative magnetic stability was evaluated from,

$$\Delta E = E_{AFM} - E_{FM}$$

where E_{FM} and E_{AFM} are the converged total energies per formula unit of the FM and AFM configurations, respectively. A positive value of ΔE indicates that the FM state is energetically preferred.

S2. Lattice-constant optimization

The equilibrium lattice constant of each LiMnTe configuration was determined from total-energy calculations performed over a series of cubic lattice constants. The α-, β-, and γ-type atomic arrangements were examined in the nonmagnetic (NM), ferromagnetic (FM), and antiferromagnetic (AFM) states. The resulting energy–volume data were expressed per formula unit and referenced to the lowest calculated energy among all considered structures,

$$E_{rel}(a) = E(a) - E_{min}$$

The resulting energy–volume data were subsequently fitted using the third-order Birch–Murnaghan equation of state[1],

$$E(V) = E_0 + \frac{9V_0B_0}{16}[(\eta - 1)^3B_0' + (\eta - 1)^2(6 - 4\eta)]$$

where

$$\eta = \left(\frac{V_0}{V}\right)^{\frac{2}{3}}$$

Here, E_0 and V_0 are the fitted equilibrium energy and volume, respectively, B_0 is the bulk modulus, and B_0' is its first pressure derivative.

S3. Analyses of structural stability

The thermodynamic stability of β-LiMnTe was examined from its formation energy relative to decomposition into bcc Li and zinc-blende (ZB) MnTe as follows[2],

$$E_{form} = E_{LiMnTe} - (E_{Li} + E_{MnTe})$$

where E_{LiMnTe}, E_{Li}, and E_{MnTe} are the total energy of LiMnTe, bcc Li, and ZB-MnTe, respectively. A negative E_{form} suggests that the material is energetically stable against decomposition into bcc Li and ZB-MnTe.

Phonon calculations were performed using the finite-displacement method implemented in PHONOPY[3, 4], with atomic forces obtained from FLEUR calculations with a $2 \times 2 \times 2$ supercell of ferromagnetic β-LiMnTe. The forces were calculated using an $8 \times 8 \times 8$ k-point mesh. The absence of imaginary frequencies indicates the structure is dynamically stable.

The elastic response of ferromagnetic β-LiMnTe was evaluated using the energy–strain method[5, 6]. Because β-LiMnTe has cubic symmetry, its second-order elastic tensor is described by three independent constants, C_{11}, C_{12}, and C_{44}. The strain parameter was varied from $\delta = -0.020$ to $+0.020$ in increments of 0.005.

For the isotropic volumetric deformation,

$$F_{bulk} = \begin{pmatrix} 1+\delta & 0 & 0 \\ 0 & 1+\delta & 0 \\ 0 & 0 & 1+\delta \end{pmatrix}$$

The energy variation is

$$\Delta E_{bulk} = \frac{9}{2} V_0 B \delta^2 + O(\delta^3)$$

where V_0 is the equilibrium volume of the cell and

$$B = \frac{C_{11} + 2C_{12}}{3}$$

is the bulk modulus.

A volume-conserving orthorhombic deformation was used to determine $C_{11} - C_{12}$:

$$F_{ortho} = \begin{pmatrix} 1+\delta & 0 & 0 \\ 0 & 1-\delta & 0 \\ 0 & 0 & \dfrac{1}{1-\delta^2} \end{pmatrix}$$

where

$$\Delta E_{ortho} = V_0 (C_{11} - C_{12}) \delta^2 + O(\delta^4)$$

The shear constant C_{44} was obtained using the volume-conserving monoclinic deformation

$$F_{shear} = \begin{pmatrix} 1 & \delta & 0 \\ \delta & 1 & 0 \\ 0 & 0 & \dfrac{1}{1-\delta^2} \end{pmatrix}$$

which gives

$$\Delta E_{shear} = 2 V_0 C_{44} \delta^2 + O(\delta^4)$$

The calculated energy–strain data were fitted to an even polynomial,

$$E(\delta) = E_0 + c_2 \delta^2 + c_4 \delta^4$$

where the quadratic coefficient $c_2$determines the corresponding elastic modulus. The independent elastic constants were then recovered from

$$C_{12} = B - \frac{C_{11} - C_{12}}{3}$$

$$C_{11} = B + \frac{2(C_{11} - C_{12})}{3}$$

The tetragonal shear modulus was defined as

$$C' = \frac{C_{11} - C_{12}}{2}$$

For an unstressed cubic crystal, the necessary and sufficient elastic stability conditions are

$$C_{11} - C_{12} > 0$$

$$C_{11} + 2C_{12} > 0$$

$$C_{44} > 0$$

These are equivalent to requiring positive values of C', B, and C_{44}. The criteria follow from the positive definiteness of the elastic-energy density and are commonly referred to as the Born–Huang mechanical stability conditions[7, 8].

The effective shear modulus was estimated using the Voigt–Reuss–Hill averaging scheme. For a cubic material, the Voigt and Reuss shear moduli are

$$G_V = \frac{C_{11} - C_{12} + 3C_{44}}{5}$$

and

$$G_R = \frac{5(C_{11} - C_{12})C_{44}}{4C_{44} + 3(C_{11} - C_{12})}$$

The Hill average[9] is

$$G = \frac{G_V + G_R}{2}$$

The Young's modulus and Poisson's ratio were subsequently calculated as

$$E = \frac{9BG}{3B + G}$$

and

$$\upsilon = \frac{3B - 2G}{2(3B + G)}$$

Pugh's ratio[10] is calculated as $\frac{B}{G}$, where values above approximately 1.75 suggest a tendency toward ductile behavior, whereas smaller values suggest brittleness.

The elastic anisotropy of the cubic phase was characterized using the Zener anisotropy factor[11],

$$A = \frac{2C_{44}}{C_{11} - C_{12}}$$

A value of $A = 1$ corresponds to an elastically isotropic cubic solid, while deviations from unity indicate directional dependence of the elastic response.

S4. Spin polarization at the Fermi level

The spin polarization at the Fermi level was evaluated from the total spin-resolved electronic density of states obtained from the collinear spin-polarized PBE calculation using a 15×15×15 *k*-point mesh. The DOS spin polarization is defined as

$$P(E_F) = \frac{N_{\uparrow}(E_F) - N_{\downarrow}(E_F)}{N_{\uparrow}(E_F) + N_{\downarrow}(E_F)} \times 100\%$$

where $N_{\uparrow}(E_{\mathrm{F}})$ and $N_{\downarrow}(E_{\mathrm{F}})$ are the total spin-up and spin-down DOS values at the Fermi level, respectively. This definition corresponds to the static DOS polarization and should be distinguished from transport spin polarization, which may additionally depend on the spin-dependent Fermi velocities, scattering rates, and experimental measurement geometry[12]. Because the assignment of spin-up and spin-down labels depends on the chosen spin-quantization axis, the magnitude $|P(E_F)|$ is reported when discussing the degree of spin polarization.

S5. Exchange coupling and Curie-temperature calculations

The magnetic exchange interactions were evaluated by mapping first-principles total energies of different collinear magnetic configurations onto a classical Heisenberg model[13].

The magnetic Hamiltonian was written as

$$\mathcal{H} = E_0 - \sum_{i<j} J_{ij} \boldsymbol{e_i} \cdot \boldsymbol{e_j}$$

where E_0 is the spin-independent contribution to the total energy, J_{ij} is the isotropic exchange interaction between Mn sites i and j, and $\mathbf{e}_i$ is a unit vector along the local Mn moment. Under this convention, $J_{ij} > 0$ favors parallel, ferromagnetic alignment, whereas $J_{ij} < 0$ favors antiparallel alignment. The exchange constants were grouped into symmetry-equivalent neighbor shells according to the Mn–Mn separation.

For magnetic configuration k, the model energy can be expressed as

$$E_k^{model} = E_0 - \sum_{n=1}^{N_j} J_n C_{kn}$$

where J_n represents the exchange constant of the n-th Mn–Mn coordination shell and

$$C_{kn} = \sum_{\langle i,j \rangle} e_i^{(k)} \cdot e_j^{(k)}$$

is the spin-correlation coefficient for that shell and configuration. The DFT total energies of four linearly independent collinear configurations constructed in a $2 \times 2 \times 2$ magnetic supercell were fitted simultaneously to obtain E_0, J_1, J_2, and J_3.

Fig. S7 presents the 2×2×1 supercell used to visualize the magnetic exchange interactions in ferromagnetic β-LiMnTe. Taking Mn0 as the reference site, J_1, J_2, and J_3 describe representative first-, second-, and third-nearest-neighbor Mn–Mn interactions at distances of 4.52, 6.39, and 7.83 Å, respectively. Only one representative pair from each coordination shell is shown, while all symmetry-equivalent interactions were included in the calculations.

The finite-temperature magnetic behavior was simulated using the VAMPIRE atomistic spin-model code[14]. VAMPIRE represents the magnetic atoms by classical unit spin vectors and allows first-principles exchange parameters to be used in Monte Carlo and spin-dynamics simulations. The exchange part of the Hamiltonian was

$$\mathcal{H}_{exc} = -\sum_{i<j} J_{ij} \boldsymbol{e_i} \cdot \boldsymbol{e_j}$$

using the same sign and pair-counting convention as in the DFT energy mapping. Particular care was taken when transferring the exchange parameters to the VAMPIRE unit-cell file to avoid an additional factor of two arising from duplicate bond counting. In the VAMPIRE convention, positive isotropic J_{ij} values favor parallel spin alignment.

Only the Mn sublattice was included as a dynamic magnetic sublattice because the DFT moments on Li and Te are negligible. Magnetocrystalline anisotropy was neglected as an approximation because the exchange interactions were expected to dominate the finite-temperature behavior. The VAMPIRE simulations therefore employed an isotropic classical Heisenberg model without an external magnetic field.

A cubic simulation box of $12 \times 12 \times 12$ nm^3 was employed with periodic boundary conditions along all three Cartesian directions to approximate bulk behavior and reduce surface effects. The temperature was varied from 0 to 1000 K in increments of 10 K. At every temperature, the system was equilibrated for 20,000 Monte Carlo steps, followed by 50,000 steps for statistical averaging.

The Curie temperature was extracted by fitting the temperature-dependent normalized magnetization obtained from the VAMPIRE Monte Carlo simulations to the critical power-law relation

$$m(T) = A\left(1 - \frac{T}{T_C}\right)^{\beta}, \qquad T < T_C$$

where $m(T) = M(T)/M(0)$, A is a fitting coefficient, T_C is the Curie temperature, and β is the magnetization critical exponent. Both T_C and β were treated as fitting parameters.

The fit was performed using data satisfying $m(T) \geq 0.10$, corresponding to the temperature range of 0–350 K. The fitting yielded $T_C = 352.28$K and a critical exponent $\beta = 0.3395$. The excellent agreement with the simulation data is indicated by $R^2 = 0.99967$ and a root-mean-square error of 0.00372. Accordingly, the estimated Curie temperature is reported as approximately 352 K.

To evaluate the finite-temperature structural stability of β-LiMnTe near its predicted Curie temperature, spin-polarized ab initio molecular dynamics simulations were performed using Vienna Ab initio Simulation Package (VASP)[15, 16] within the Projector augmented-wave (PAW) framework[17] and PBE functional[18]. A 2×2×2 replication of the conventional cell, containing 96 atoms, was simulated in the canonical NVT ensemble at 350 K using a Nosé–Hoover thermostat[19, 20]. The Brillouin zone was sampled at the Γ point only. A plane-wave cutoff energy of 450 eV and an electronic convergence criterion of 10^{-6} eV were employed. The equations of motion were integrated using a time step of 1 fs for 10,000 steps, corresponding to a total simulation time of 10 ps. As shown in Fig. S8, the final AIMD configuration retains the overall crystal framework but exhibits noticeable thermal displacements of the Mn atoms from their ideal lattice sites. This Mn-sublattice distortion is accompanied by a modest reduction in the total magnetic moment, suggesting a coupling between thermally induced structural distortions and the weakening of ferromagnetism near the predicted T_C.

S6. Anomalous and spin Hall transport calculations

The intrinsic anomalous Hall conductivity (AHC) and spin Hall conductivity (SHC) were calculated using a Wannier-interpolation approach. Fully relativistic spinor wave functions were first obtained from FLEUR calculations including spin–orbit coupling. The magnetization and spin-quantization axes were chosen along the crystallographic $z \parallel [001]$ direction. The spin–orbit-coupled Bloch states were calculated on a uniform $15 \times 15 \times 15$ k-point mesh using the converged charge density of the ferromagnetic ground state.

Maximally localized Wannier functions were constructed using Wannier90[21]. Initial projections were chosen from the orbitals contributing most strongly near the Fermi level, namely Li-s, Mn-s,p,d, and Te-s,p, resulting in a total of 28 spinor Wannier functions. The frozen inner window was set to 5 eV above E_F. For entangled bands, the disentanglement procedure was used to identify a smooth low-energy subspace before minimizing the real-space Wannier spreads.

The quality of the Wannier representation was verified by comparing the Wannier-interpolated bands with the direct spin–orbit-coupled DFT bands along the selected high-symmetry path. Good agreement was obtained throughout the energy region relevant to the Hall calculations, especially near the Fermi level as seen in Fig. S2.

The intrinsic AHC was evaluated from the Brillouin-zone integral of the occupied-state Berry curvature using WannierBerri[22]. For the selected magnetization direction, the relevant conductivity component is σ_{xy}, expressed as[23]

$$\sigma_{xy} = -\frac{e^2}{\hbar}\sum_{n}\int_{BZ}\frac{d^3\boldsymbol{k}}{(2\pi)^3} f_{n\boldsymbol{k}}\Omega_{n,z}(\boldsymbol{k})$$

where $f_{n\boldsymbol{k}}$ is the occupation factor and

$$\Omega_{n,z}(\boldsymbol{k}) = -2\mathrm{Im}\left\langle \frac{\partial u_{n\boldsymbol{k}}}{\partial k_x} \middle| \frac{\partial u_{n\boldsymbol{k}}}{\partial k_y} \right\rangle$$

is the z-component of the band-resolved Berry curvature. The intrinsic AHC obtained in this way corresponds to the clean-limit Berry-curvature contribution; disorder-dependent skew-scattering and side-jump terms were not included. The use of Wannier interpolation for AHC calculations follows the formalism of Wang *et al.*[23] and its high-performance implementation in WannierBerri.

The intrinsic SHC was calculated using the Wannier-interpolation formalism of Ryoo, Park, and Souza[24]. The component σ_{xy}^{z} describes a spin current flowing along x, polarized along z, in response to an electric field along y. In the static clean limit, it was evaluated from the Kubo expression

$$\sigma_{xy}^{z} = \frac{e}{\hbar}\int_{BZ}\frac{d^3\boldsymbol{k}}{(2\pi)^3}\sum_{n}\sum_{m\neq n}(f_{n\boldsymbol{k}} - f_{m\boldsymbol{k}})\frac{\mathrm{Im}\left[\langle u_{n\boldsymbol{k}}|\hat{J}_x^z|u_{m\boldsymbol{k}}\rangle\langle u_{m\boldsymbol{k}}|\hat{v}_y|u_{n\boldsymbol{k}}\rangle\right]}{(\varepsilon_{n\boldsymbol{k}} - \varepsilon_{m\boldsymbol{k}})^2}$$

where $\hat{v}_y$ is the velocity operator and the conventional spin-current operator is

$$\hat{J}_x^z = \frac{1}{2}\{\hat{s}_z, \hat{v}_x\}$$

The Hall conductivities were integrated over the full three-dimensional Brillouin zone using a uniform $200 \times 200 \times 200$ k-point mesh. To identify the electronic states responsible for the Hall responses, the band-resolved Berry curvature $\Omega_{n,\boldsymbol{k}}$, and spin Berry curvature $\Omega_{n,xy}^{z}(\boldsymbol{k})$ were evaluated along the selected high-symmetry path. The occupied-state sums $\sum_n f_{n\boldsymbol{k}}\Omega_{n,z}(\boldsymbol{k})$ and $\sum_n f_{n\boldsymbol{k}}\Omega_{n,xy}^{z}(\boldsymbol{k})$ were also calculated to visualize the sign and relative strength of the local contributions. These high-symmetry-path plots were used only to interpret the microscopic origin of the Hall effects, while the reported AHC and SHC values were obtained from integration over the full Brillouin zone.

Supporting Figures

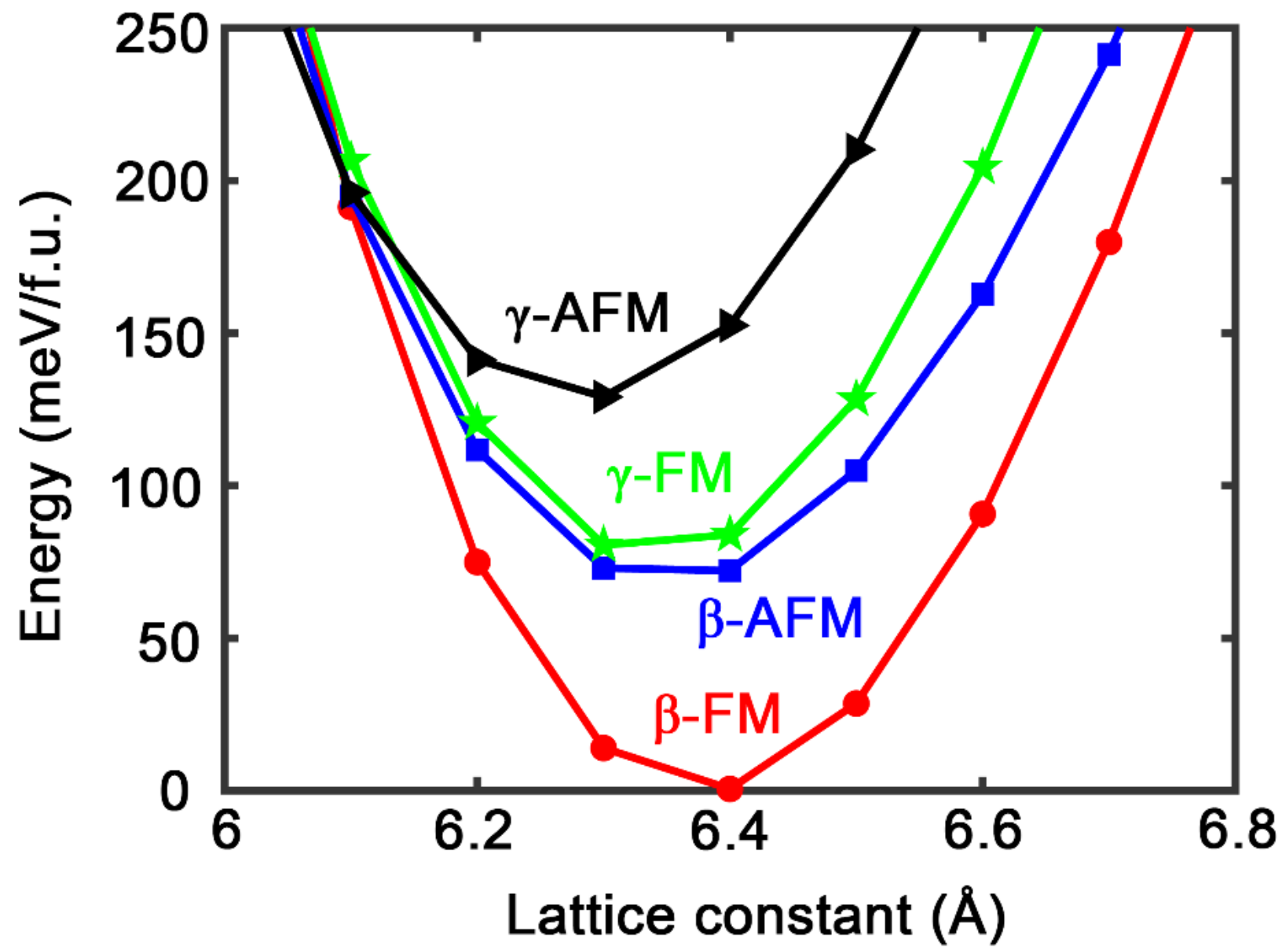

Fig. S1 Magnified total-energy comparison of the magnetic configurations of LiMnTe as a function of lattice constant. The energy is referenced to the minimum energy of the β-FM phase, confirming β-FM LiMnTe as the lowest-energy configuration among those considered.

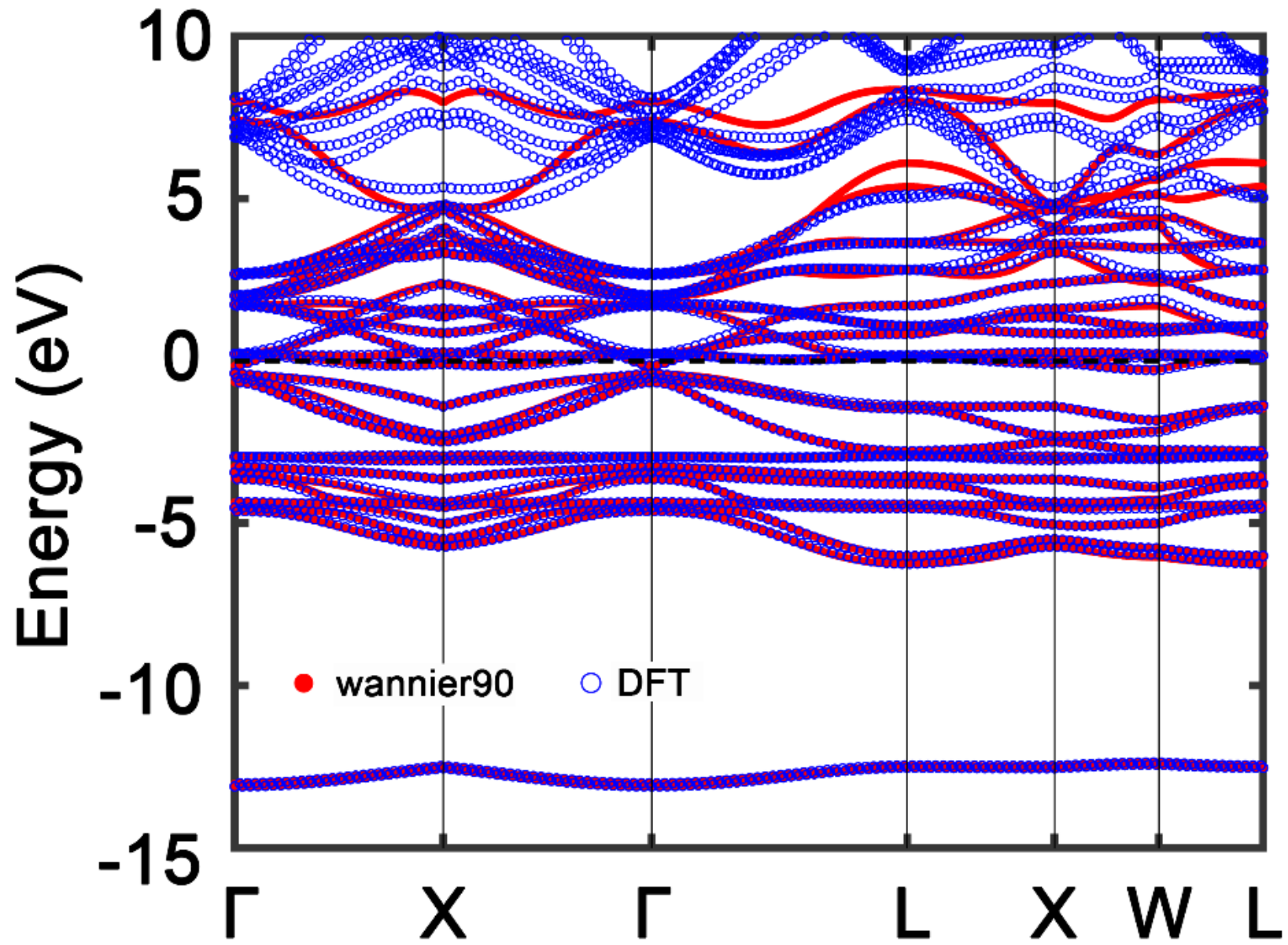

Fig. S2 Comparison between spin-orbit-coupled DFT and Wannier90-interpolated band structures of ferromagnetic β-LiMnTe. The blue open circles and red filled circles represent the DFT and Wannier90 results, respectively. The Fermi level is set to zero.

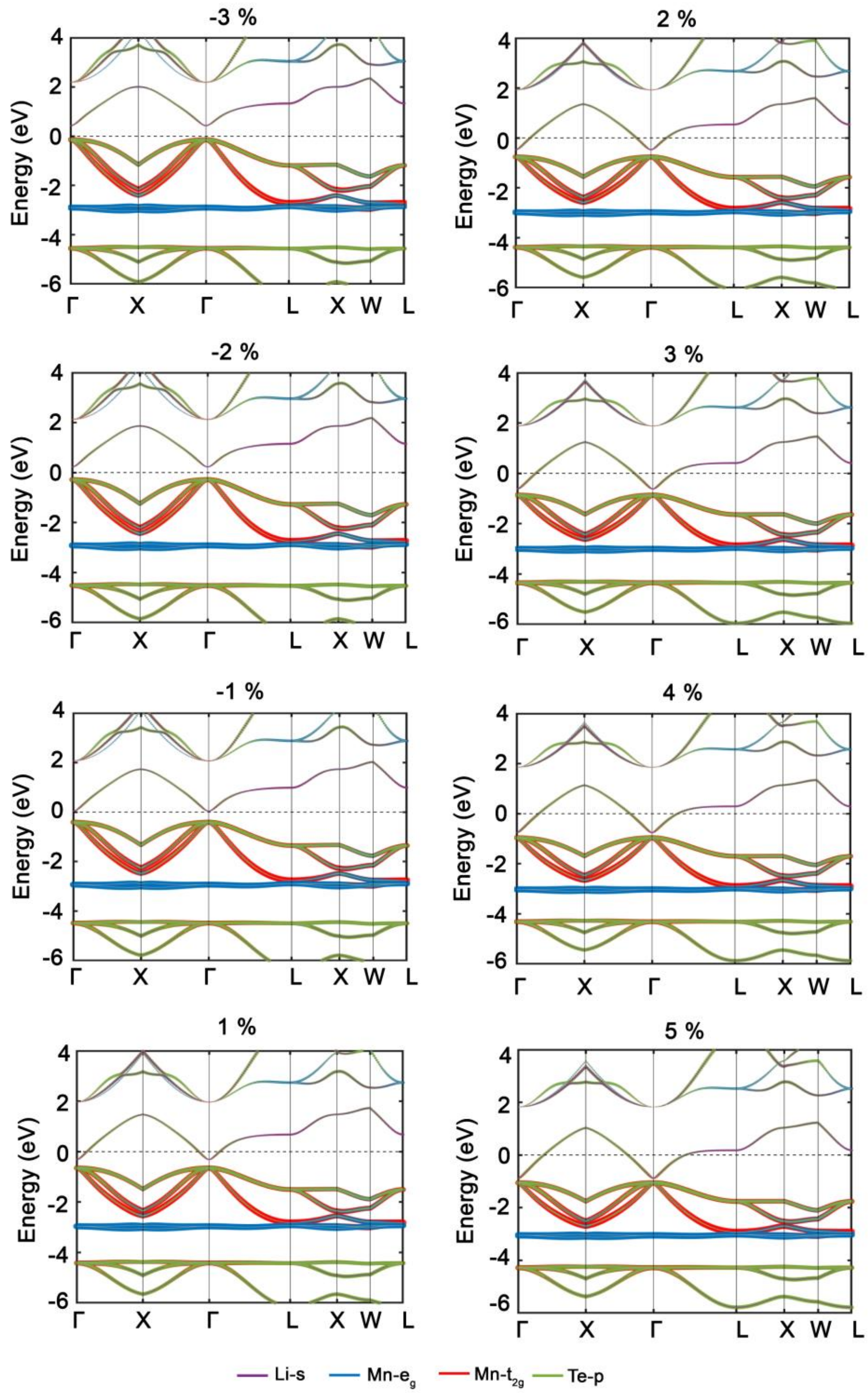

Fig. S3 Spin-up orbital-projected band structures of ferromagnetic β-LiMnTe under selected nonzero isotropic volumetric strain from -3% to +5%. The Fermi level is set to zero. The colors indicate the dominant orbital contributions from Li-s, Mn-e_g, Mn-t_{2g}, and Te-p states.

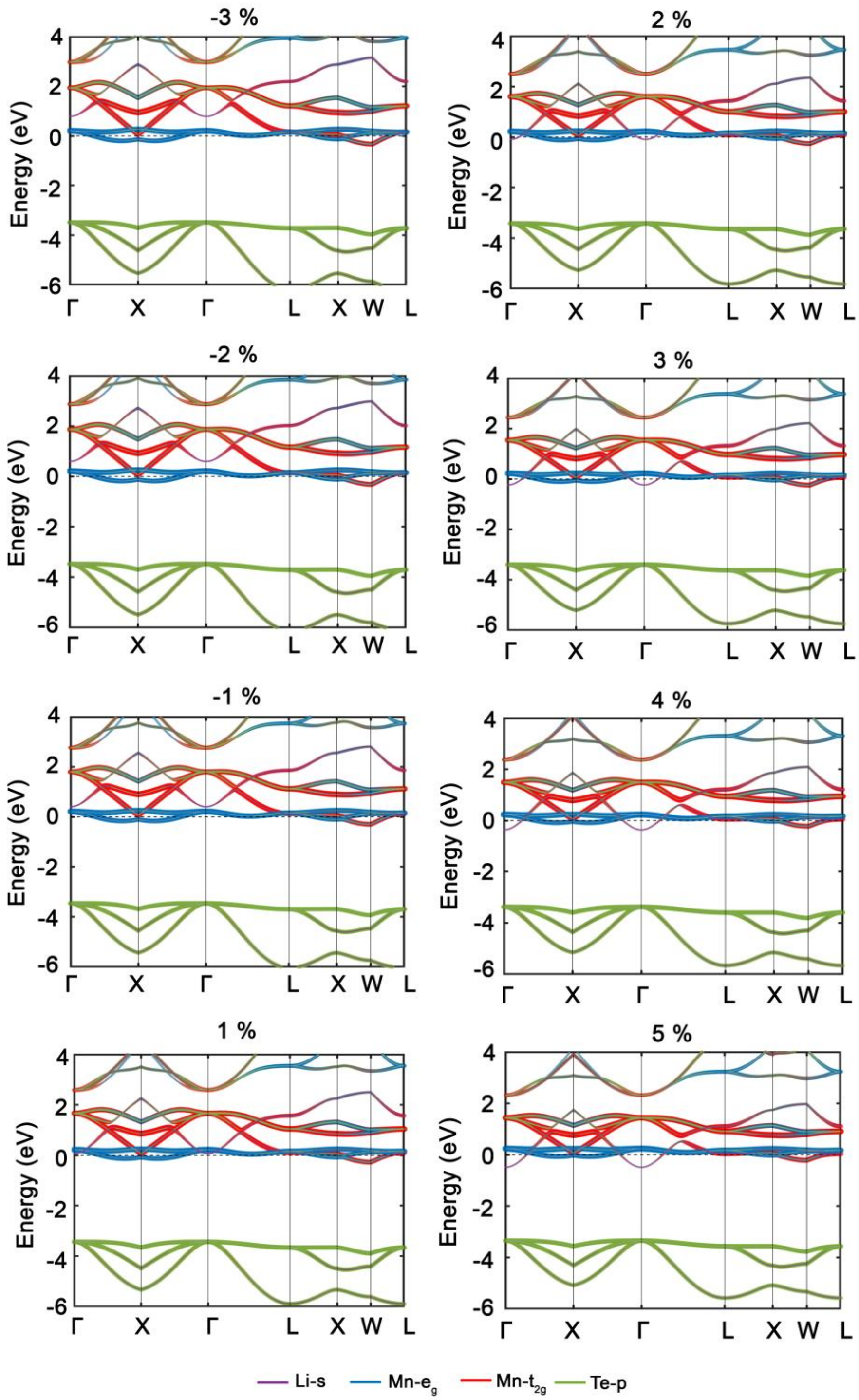

Fig. S4 Spin-down orbital-projected band structures of ferromagnetic β-LiMnTe under selected nonzero isotropic volumetric strain from -3% to +5%. The Fermi level is set to zero. The colors indicate the dominant orbital contributions from Li-s, Mn-e_g, Mn-t_{2g}, and Te-p states.

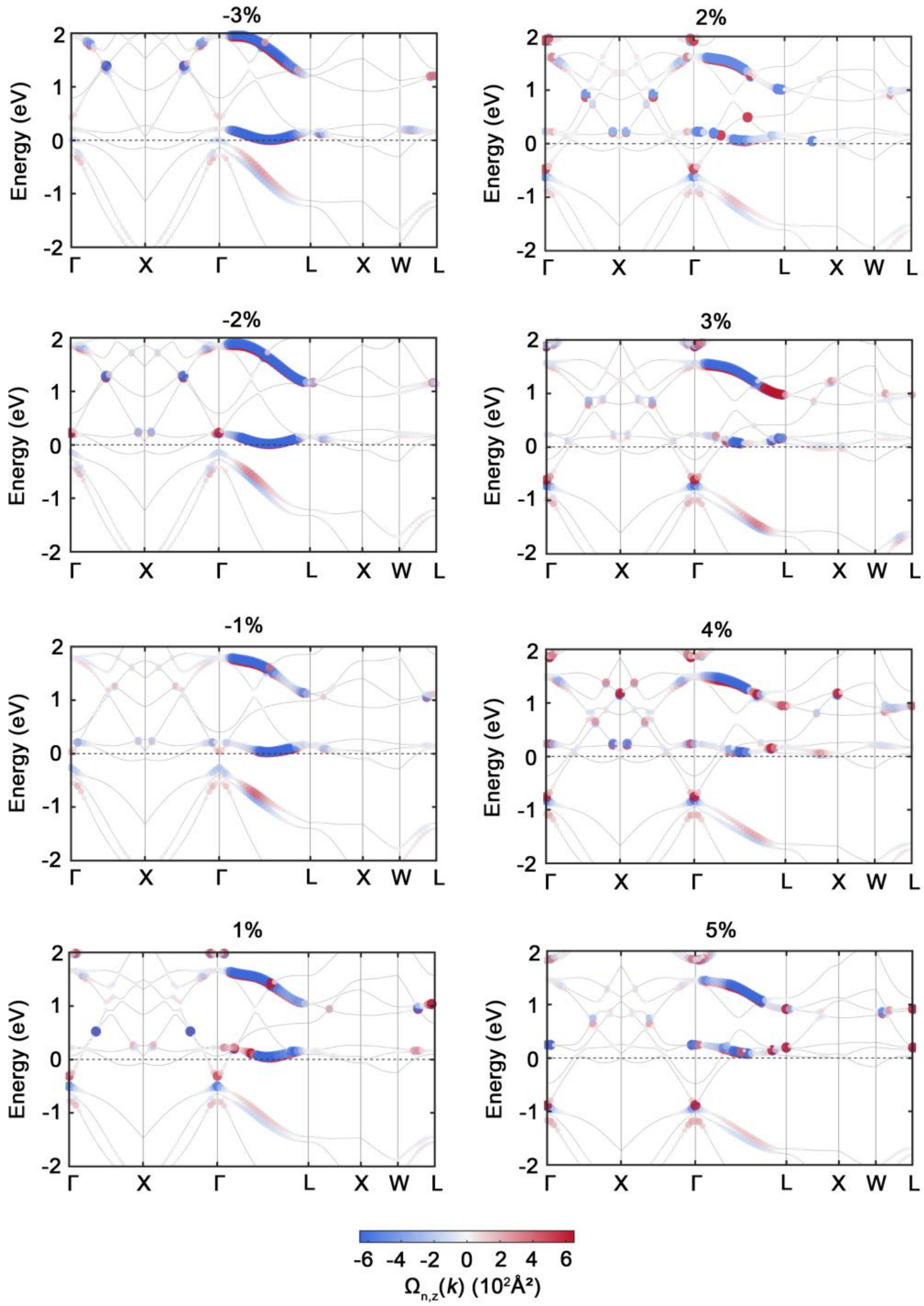

Fig. S5 Berry-curvature-resolved band structures of ferromagnetic β-LiMnTe under selected nonzero isotropic volumetric strain from −3% to +5%. The color scale represents the band-resolved Berry curvature $\Omega_{n,z}(\mathbf{k})$, where red and blue indicate positive and negative contributions, respectively. Fermi level is set to zero.

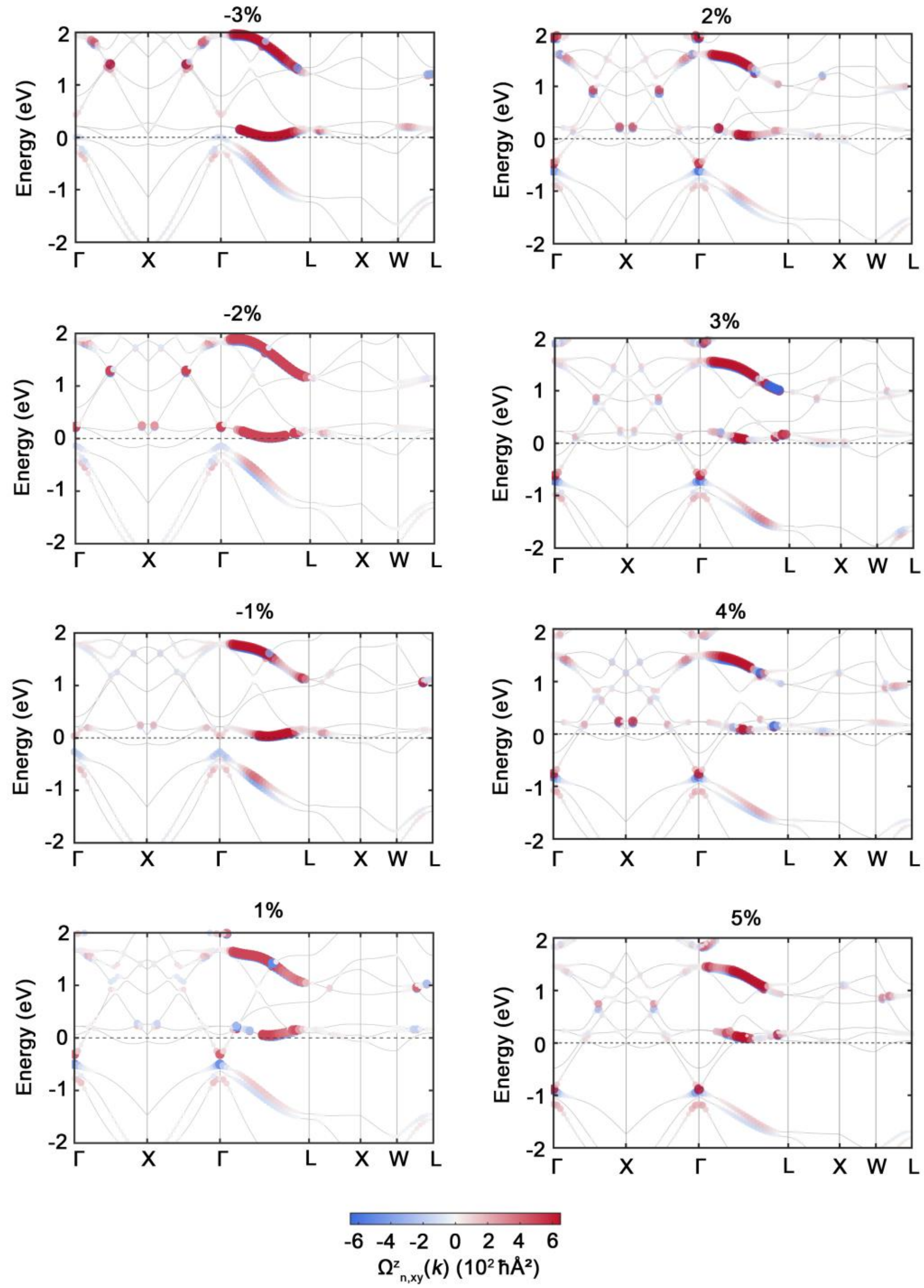

Fig. S6 Spin-Berry-curvature-resolved band structures of ferromagnetic β-LiMnTe under selected nonzero isotropic volumetric strain from -3% to $+5\%$. The color scale represents the band-resolved spin Berry curvature $\Omega^{z}_{n,xy}(\mathbf{k})$, where red and blue indicate positive and negative contributions, respectively. Fermi level is set to zero.

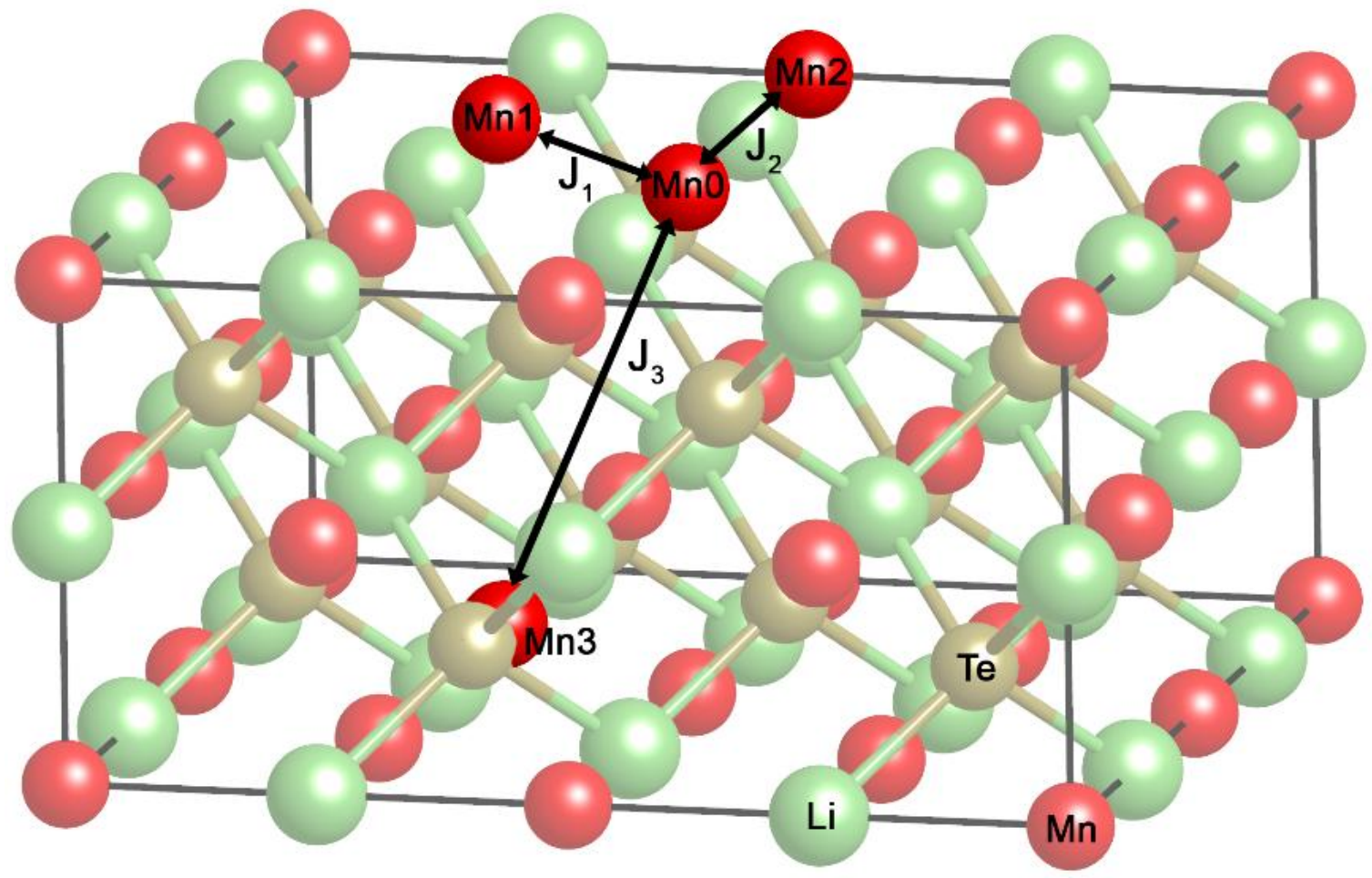

Fig. S7. 2×2×1 supercell of ferromagnetic β-LiMnTe used for the exchange-interaction visualization. Li, Mn, and Te atoms are shown in green, red, and gold, respectively. A reference Mn site is connected to representative first-, second-, and third-nearest-neighbor Mn sites corresponding to J_1, J_2, and J_3. The respective Mn–Mn distances are 4.52, 6.39, and 7.83 Å. Only one representative interaction from each coordination shell is displayed for clarity.

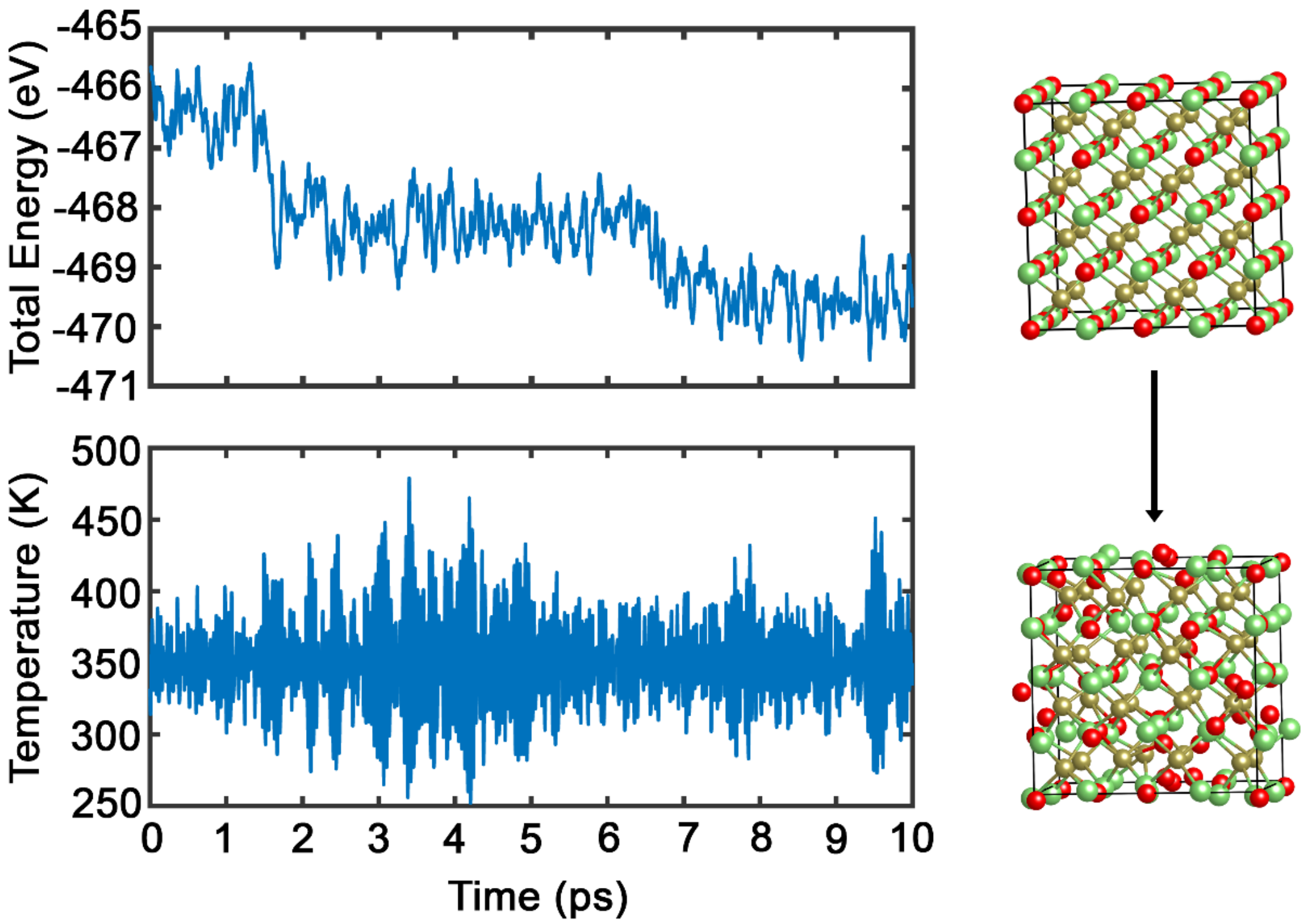

Fig. S8. Time evolution of the total energy and temperature of β-LiMnTe during a 10 ps NVT AIMD simulation at 350 K, together with the initial and final configurations. The overall half-Heusler framework is retained, although the final configuration exhibits noticeable thermally induced distortion of the Mn sublattice, accompanied by a modest reduction in the total magnetic moment.

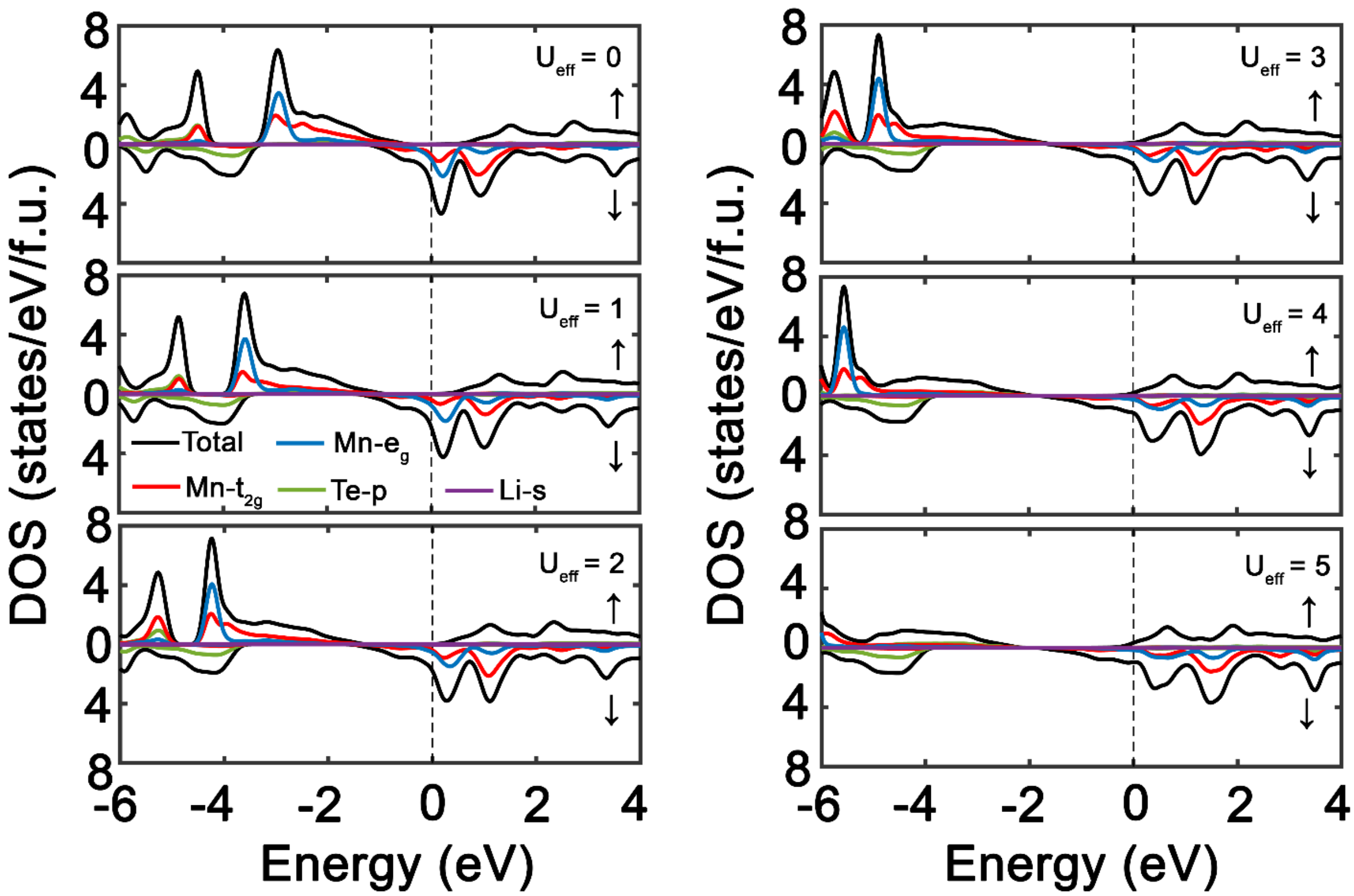

Fig. S9. Spin-resolved total and orbital-projected densities of states (DOS) of ferromagnetic β-LiMnTe calculated using DFT+U with U_{eff} = 0 – 5 eV applied to the Mn-3d orbitals. The total DOS and the Mn-e_g, Mn-t_{2g}, Te-p, and Li-s contributions are shown in black, blue, red, green, and purple, respectively. The Fermi level is set to zero.

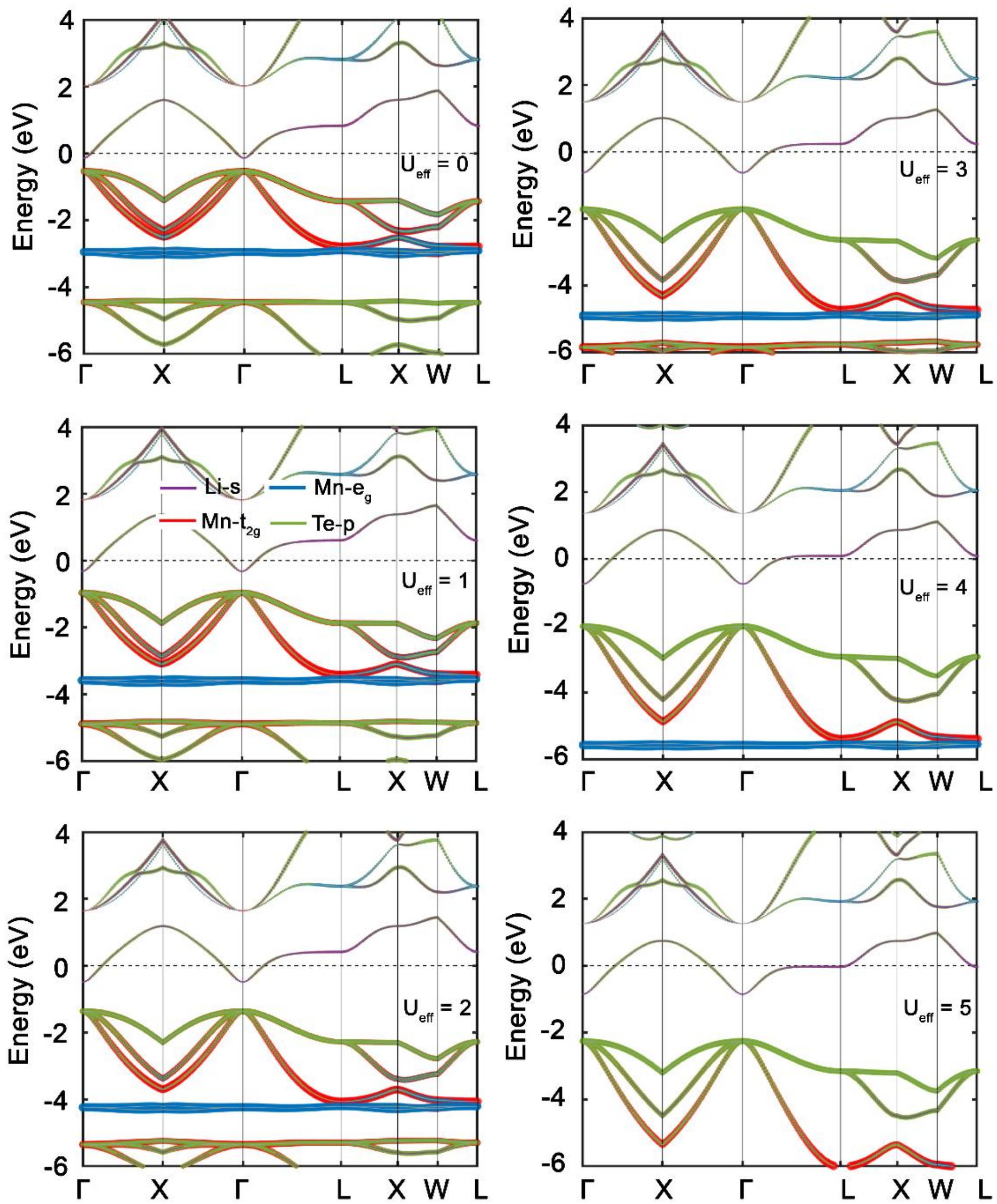

Fig. S10. Orbital-resolved spin-up band structures of ferromagnetic β-LiMnTe calculated using DFT+U with U_{eff} = 0 – 5 eV applied to the Mn-3d orbitals. The Li-s, Mn-t_{2g}, Mn-e_g, and Te-p contributions are shown in purple, red, blue, and green, respectively. The Fermi level is set to zero.

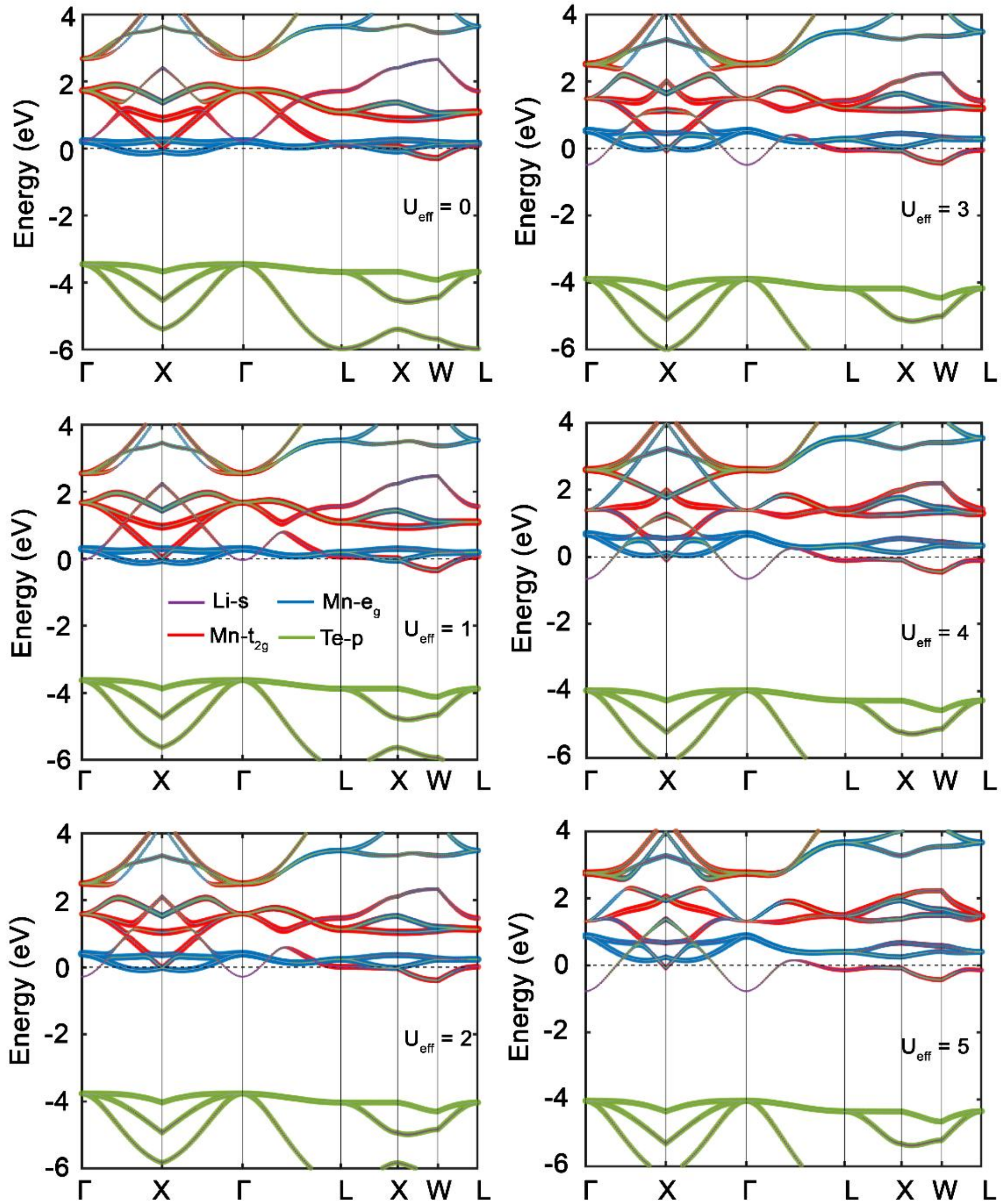

Fig. S11. Orbital-resolved spin-down band structures of ferromagnetic β-LiMnTe calculated using DFT+U with U_{eff} = 0 – 5 eV applied to the Mn-3d orbitals. The Li-s, Mn-t_{2g}, Mn-e_g, and Te-p contributions are shown in purple, red, blue, and green, respectively. The Fermi level is set to zero.

References

1. F. Birch, Phys. Rev., 1947, 71, 809-824.
2. T. T. Hoang, S. H. Rhim and S. C. Hong, Phys. Rev. Mater., 2022, 6, 055001.
3. A. Togo and I. Tanaka, Scr. Mater., 2015, 108, 1-5.
4. A. Togo, L. Chaput, T. Tadano and I. Tanaka, J. Phys.: Condens. Matter, 2023, 35, 353001.
5. M. J. Mehl, J. E. Osburn, D. A. Papaconstantopoulos and B. M. Klein, Phys. Rev. B, 1990, 41, 10311-10323.
6. P. Ravindran, L. Fast, P. A. Korzhavyi, B. Johansson, J. Wills and O. Eriksson, J. Appl. Phys., 1998, 84, 4891-4904.
7. M. Born and K. Huang, *Dynamical Theory Of Crystal Lattices*, Oxford University Press, 1996.
8. F. Mouhat and F.-X. Coudert, Phys. Rev. B, 2014, 90, 224104.
9. R. Hill, Proc. Phys. Soc. Sec. A, 1952, 65, 349.
10. S. F. Pugh, Lond. Edinb. Dublin Philos. Mag. J. Sci., 1954, 45, 823-843.
11. C. M. Zener and S. Siegel, J. Phys. Colloid Chem., 1949, 53, 1468-1468.
12. I. I. Mazin, Phys. Rev. Lett., 1999, 83, 1427-1430.
13. G. S. Joyce, Phys. Rev., 1967, 155, 478-491.
14. R. F. L. Evans, W. J. Fan, P. Chureemart, T. A. Ostler, M. O. A. Ellis and R. W. Chantrell, J. Phys.: Condens. Matter, 2014, 26, 103202.
15. G. Kresse and J. Hafner, Phys. Rev. B, 1993, 47, 558-561.
16. G. Kresse and J. Furthmüller, Phys. Rev. B, 1996, 54, 11169-11186.
17. P. E. Blöchl, Phys. Rev. B, 1994, 50, 17953-17979.
18. J. P. Perdew, K. Burke and M. Ernzerhof, Phys. Rev. Lett., 1996, 77, 3865-3868.
19. S. Nosé, J. Chem. Phys., 1984, 81, 511-519.
20. W. G. Hoover, Phys. Rev. A, 1985, 31, 1695-1697.
21. G. Pizzi, V. Vitale, R. Arita, S. Blügel, F. Freimuth, G. Géranton, M. Gibertini, D. Gresch, C. Johnson, T. Koretsune, J. Ibañez-Azpiroz, H. Lee, J.-M. Lihm, D. Marchand, A. Marrazzo, Y. Mokrousov, J. I. Mustafa, Y. Nohara, Y. Nomura, L. Paulatto, S. Poncé, T. Ponweiser, J. Qiao, F. Thöle, S. S. Tsirkin, M. Wierzbowska, N. Marzari, D. Vanderbilt, I. Souza, A. A. Mostofi and J. R. Yates, J. Phys.: Condens. Matter, 2020, 32, 165902.
22. S. S. Tsirkin, npj Comput. Mater., 2021, 7, 33.
23. X. Wang, J. R. Yates, I. Souza and D. Vanderbilt, Phys. Rev. B, 2006, 74, 195118.
24. J. H. Ryoo, C.-H. Park and I. Souza, Phys. Rev. B, 2019, 99, 235113.
